# Decoding the Structure of Non-Proteinogenic Amino Acids: The Rotational Spectrum of Jet-Cooled Laser-Ablated Thioproline

**DOI:** 10.3390/molecules26247585

**Published:** 2021-12-14

**Authors:** Juan Carlos López, Alberto Macario, Andrés Verde, Alfonso Pérez-Encabo, Susana Blanco

**Affiliations:** 1 Departamento de Química Física y Química Inorgánica, Facultad de Ciencias, IU CINQUIMA Universidad de Valladolid, 47011 Valladolid, Spain; juancarlos.lopeza@uva.es (J.C.L.); or alberto.macario@univ-rennes1.fr (A.M.); andres.verde@alumnos.uva.es (A.V.); 2 Departamento de Química Orgánica, Facultad de Ciencias, Universidad de Valladolid, 47011 Valladolid, Spain; alfonso.perez.encabo@uva.es

**Keywords:** laser-ablation, rotational spectroscopy, amino acids, computational chemistry, intramolecular interactions

## Abstract

The broadband rotational spectrum of jet-cooled laser-ablated thioproline was recorded. Two conformers of this system were observed and identified with the help of DFT and ab initio computations by comparison of the observed and calculated rotational constants and ^14^N quadrupole coupling constants as well as the predicted energies compared to the observed relative populations. These conformers showed a mixed bent/twisted arrangement of the five-membered ring similar to that of the related compound thiazolidine with the N–H bond in axial configuration. The most stable form had the COOH group in an equatorial position on the same side of the ring as N-H. The arrangement of the C=O group close to the N-H bond led to a weak interaction between them (classified as type I) characterized by a noncovalent interaction analysis. The second form had a *trans*-COOH arrangement showing a type II O–H···N hydrogen bond. In thioproline, the stability of conformers of type I and type II was reversed with respect to proline. We show how the conformation of the ring depends on the function associated with the endocyclic N atom when comparing the structures of isolated thioproline with its zwitterion observed in condensed phases and with peptide forms.

## 1. Introduction

Protein activity is a consequence of the three-dimensional arrangement of their different functional groups. For example, the activity of folded proteins relies on factors as the secondary structure formation, the hydrophobic effect, and additional noncovalent interactions to organize functional groups to allow molecular recognition of specific substrate partners and catalysis. Enhanced activity and expanding functions of proteins may be incorporated through the introduction of amino acids beyond the natural ones, for example, amino acids restrained conformationally, incorporating new functionalities, or enhancing hydrophobic effect [1,2,3]. The field of protein design and engineering aims to achieve the functions of proteins in novel structures and different developments.

Proline residues are unique among the canonical amino acids, due to the conformational restraint of backbone cyclization and the presence of a tertiary amide bond [4]. These structural characteristics limit the available conformations for its residues, which enables proline to be preferentially observed in specific structural contexts, such as secondary structure termination, loops, turns, and helices [5]. Proline residues also present distinctive biomolecular recognition properties due to their differences from other canonical amino acids [6]. The importance of proline derivatives can be exemplified by the role of 4R-hydroxyproline, also studied in isolation [7], in the stabilization of collagen [8,9].

**Scheme 1:**
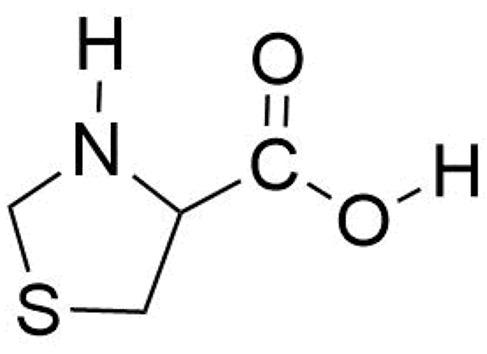
The thioproline (SPro) molecule.

Thioproline (thiazolidine-4-carboxylic acid, SPro, see Scheme 1) is a sulfur-containing α-amino acid analog of proline for which the methylene group at position 4 of the pyrrolidine ring of proline is replaced by a sulfur atom to give a thiazolidine ring. It is well known that SPro is produced when cysteine (Cys) condenses with reactive formaldehyde [10]. Thiazolidines exhibit a wide variety of pharmaceutical properties and SPro in particular has attracted interest for its antioxidant properties in diverse areas of medicine [11,12,13,14,15,16] and has demonstrated to be an effective endogenous nitric-oxide-trapping agent [17,18,19]. It has been shown that oxidative stress to a cell could induce protein post-translational modification (PTM) in which proteic cysteine would react with formaldehyde generated by oxidants added to the medium to form proteic SPro [20,21]. This reaction has been used to quantify oxidizing agent-exposed *Escherichia coli* samples and to prove that SPro residues could have been an antioxidative survival mechanism that further protect cells [22]. It has also been found that SPro could also be introduced into proteins via misincorporation of SPro in place of proline during protein synthesis [23]. SPro forms part of the structure of kynostatin 272 (KNI-272) a pentapeptide mimic containing two sulfur atoms that acts as a conformationally constrained inhibitor of HIV protease [24,25,26,27,28] and for which the role of the SPro residue in the conformation has been discussed [26,27]. In the same context, it forms part of the structure of possible prolyl-oligopeptidase inhibitors of use in the prevention of Alzheimer’s disease and senile dementia [29]. 

SPro as generally occur for amino acids crystalizes in its zwitterionic form and the studies of its structure by X-ray diffraction yield to a single form with the carboxyl group adopting an equatorial arrangement and the ring adopting an S_γ_-*endo* bent form [30]. The conformation adopted by SPro residues in KNI-272 for example was discussed based on two possible *endo* or *exo* forms of the ring and the *cis-trans* amide bonds equilibrium [26,27], as occur for Pro [4] residues. The same equilibria reinforced by hydrogen bonding between the acid and imino group exists in isolated Pro leading to four conformers [31,32]. It is thus interesting to characterize the structure of SPro in isolation in the gas phase to better understand its behavior, to analyze its stable conformations, their energies, and the intramolecular interactions contributing to stabilizing them.

The combination of Fourier transform microwave (FTMW) spectroscopies with supersonic-jet expansion techniques has contributed to a new era for rotational spectroscopy which can be considered as the most definitive gas-phase structural probe [33,34,35,36]. It makes possible the unambiguous discrimination between different isomers, including tautomers, conformers, or isotopomers. The spatial mass distribution of such species has unique spectroscopic constants and distinct individual rotational spectra. In addition, the quadrupole coupling hyperfine structure due to the presence of the ^14^N gives precise information of the orientation of the amino group that helps to assign conformers with similar rotational constants. Thus, these key features make this technique to be a powerful tool. Challenging molecular systems including solid biomolecules are now accessible to this technique by using laser ablation to vaporize the samples [37,38]. In this work, we present the study of the broadband rotational spectrum of laser-ablated SPro to characterize its most stable conformers and their properties.

## 2. Results and Discussion

### 2.1. Conformational Panorama

**Figure 1:**
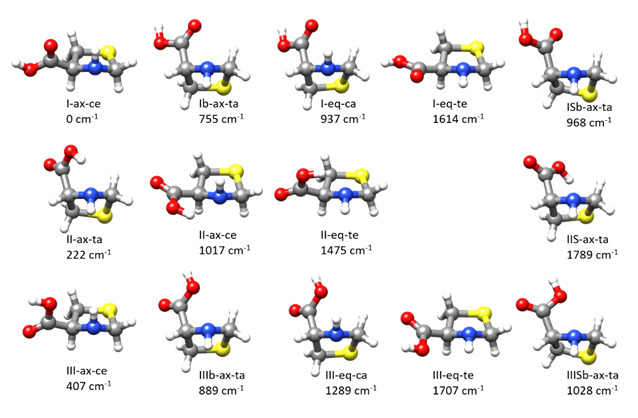
The different conformers found for thioproline from exploration of the potential energy surface. The labeling is described in the text. The quoted relative energies were calculated at the B3LYP-D3/6-311++G(2d,p) level (see Figure S2 for other perspectives).

As occur for other amino acids [31,32,37] SPro has a complex conformational panorama due to the flexibility arising from the ring-puckering vibrations of the thiazolidine ring, the N–H inversion, and the rotation of the carboxylic group around the C–C bond. Thiazolidine ring adopts bent forms with some degree of twisting with the N–H group out of the ring plane in axial or equatorial forms (see Figure S1) and two equivalent minima for each conformer. For this molecule, the axial form is the global minimum and the only conformer observed experimentally [39]. The search of the different stable conformers using CREST (conformer-rotamer ensemble sampling tool) [40] followed by B3LYP-D3/6-311++G(2d,p) optimizations lead to a total of 14 conformers given in Figure 1 (see also Figure S2 which shows different orientations). We have labeled the conformers with three symbols X-Y-Z referring to the classification of these conformers according to three structural features. X refers to the possible hydrogen bond (HB) interactions between the different polar groups. As in other amino acids [37], the interactions between the acid and amino groups are classified as type I forms when there is an N–H···O=C bond, type II when an O–H···N HB, with *trans*-COOH configuration, is established and type III when the HB is N–H···O-H. In the present case, an additional HB of the type O–H···S is possible which we have called type IIS. In conformers where there are no HBs, we have used the notations Ib when the C=O group is close to the N atom, and IIIb when the OH group, in a *cis*-COOH configuration, is close to nitrogen. In the case where the *trans*-COOH group is aligned with the S atom, we have used ISb if the C=O group points to S and IIISb when the O–H group is closer to the S atom. The second feature is thiazolidine fragment configuration which we have classified as *axial* (Y = ax) or *equatorial* (Y = eq) (see Figure S1). The third feature is the orientation of the acid group. It can be classified as axial or equatorial but to avoid confusion with the orientation of the N–H group we have chosen to use also the possible arrangements as a function of the *cis* or *trans* orientation of the COOH group relative to the side of the ring of the N–H group. In this way, we can have *trans*-axial (Z = ta), *trans*-equatorial (Z = te), *cis*-axial (Z = ca), or *cis*-equatorial (Z = ce) orientations of the COOH group (see Figure S1).

The three lowest energy conformers present, respectively, type I, II, and III interactions. The global minimum I-ax-ce has an arrangement of the amino and acid groups that may give rise to an N–H···O=C HB. It has an axial N–H group and an equatorial COOH group both in a *cis* arrangement. The next conformer in order of energy is II-ax-ta, having an O–H···N HB, both N–H and COOH groups in axial configurations but in a *trans* mutual arrangement. The third conformer in order of energy, III-ax-ce, is similar to the most stable form but with the COOH group rotated by ca. 180° so that it may give rise to a type III N–H···O–H interaction. The calculated B3LYP-D3/6-311++G(2d,p) energies, the rotational and quadrupole coupling parameters for the predicted conformers are given in Table S1. The same data for the low energy conformers were also calculated at MP2/6-311++G(2d,p) giving good predictions of the rotational and quadrupole coupling constants. The Gibbs energies and the centrifugal distortion constants were also predicted at this level from harmonic force field calculations. The results are collected in Table 1 and Table S2.

### 2.2. Rotational Spectra

**Figure 2:**
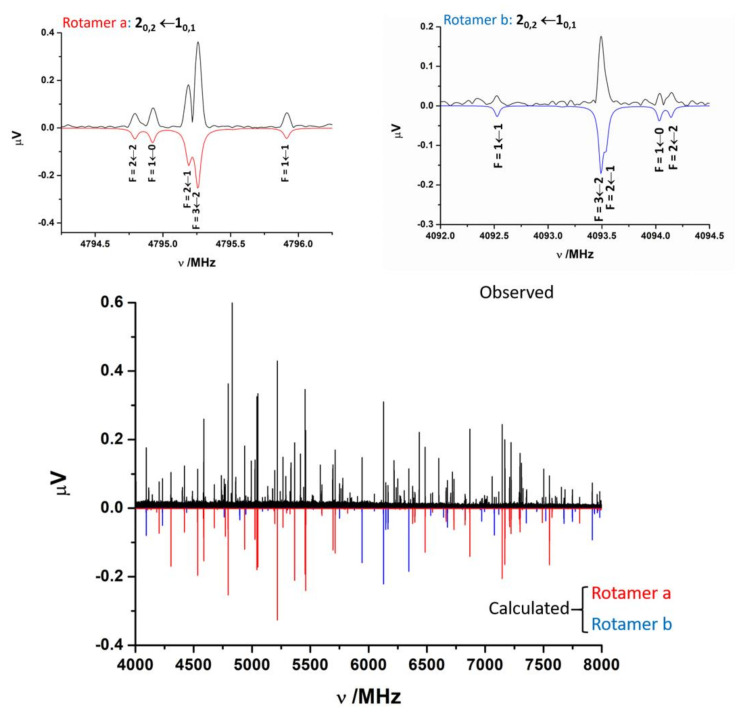
The 4–8 GHz CP-FTMW spectrum of laser-ablated SPro is shown. Two rotamers *a* and *b* of thioproline have been identified. The spectra calculated from the experimentally determined rotational parameters are shown below, in red for rotamer *a* and in blue for rotamer *b*. The figure shows also in detail the hyperfine F′←F″ components associated to the 2_0,2_←1_0,1_ transition for both rotamers. Besides the rotational parameters the ^14^N hyperfine structure, markedly different for these rotamers, is a key for the identification of the different conformers.

The jet-cooled rotational spectrum of laser-ablated SPro is shown in Figure 2. The SPro conformers are all prolate asymmetric rotors with values of the Ray asymmetry parameter *κ* between −0.75 and −0.95. First trials to assign the spectra of SPro rotamers were focussed on those conformers having a non-zero value of the *μ*_a_-electric dipole component. The *μ*_a_-type R-branch rotational lines for near prolate rotors appear to form groups regularly spaced at frequency intervals equal to (*B*+*C*). Two rotamers initially labeled *a* and *b* could be identified based on these patterns. The lines corresponding to these rotamers show the quadrupole coupling hyperfine structure due to the presence in the molecule of one nitrogen atom (see Figure 2). In both cases, an iterative procedure of measurement and prediction leads to the assignment of the complete spectrum. Rotamer *a*, which corresponds to the most intense *μ*_a_-type spectrum, shows also intense *μ*_b_- and *μ*_c_-type spectra. Rotamer *b* shows weak *μ*_b_- and *μ*_c_-type spectra. Once cleaning the spectrum from the assigned spectra, no other rotamer attributable to SPro was identified. The observed spectra do not have a sufficient signal-to-noise ratio to observe ^34^S, ^13^C, or ^14^N isotopologues in natural abundance.

The analysis [41] of the measured spectra was performed using a Hamiltonian including semirigid rotor (*H*_R_^S^) [42] and quadrupole coupling *H*_Q_ [43] terms. The semirigid rotor Hamiltonian was set up in the Watson’s S-reduction [42] and I^r^ representation:

(1) $$H_{R}^{S}=AP_{a}^{2}+BP_{b}^{2}+CP_{c}^{2}-D_{J}P^{4}-D_{JK}P^{2}P_{a}^{2}-D_{K}P_{a}^{4}+d_{1}P^{2}\left(P_{+}^{2}+P_{-}^{2}\right)+d_{2}\left(P_{+}^{4}+P_{-}^{4}\right)$$

The spectroscopic parameters associated are the rotational constants *A*, *B*, and *C*, and quartic centrifugal distortion constants, *D*_J_, *D*_JK_, *D*_K_, *d*_1,_ and *d*_2_. The quadrupole coupling interaction arises from the interaction of the nuclear electric quadrupole moment (eQ) of the ^14^N atom with the electric field gradient (*q*_αβ_ = ∂^2^V/∂α∂β, α,β = *a*,*b*,*c*) created by the rest of molecular charges at the site of this nucleus. As a consequence of this interaction, the nuclear spin of ^14^N (**I** = 1) couples to the overall rotation angular momentum (**J**) to form a resultant **F** (**F = I+J**). The determinable spectroscopic parameters are the elements of the nuclear quadrupole coupling tensor ***χ***, linearly related to the electric field gradient tensor ***q*** by ***χ*** = e*Q**q***. Usually, for ^14^N, only the diagonal elements of this tensor (*χ*_aa_, *χ*_bb_, and *χ*_cc_) are determined. The experimental parameters obtained from the fit of the observed frequencies to this Hamiltonian are shown in Table 1. The spectra simulated with the fitted parameters are compared to the observed CP-FTMW spectrum in Figure 2. The spectrum for rotamer *a* is drawn in red and that which corresponds to rotamer *b* in blue. The frequencies of the observed transitions are gathered in Tables S4 and S5 of the Supporting Information. 

### 2.3. Conformer Assignment

The comparison of the observed parameters with those predicted from theoretical calculations shown in Table S1 allows the identification of the observed rotamers. From the predicted structures and the corresponding rotational parameters, we may distinguish three families. Those conformers having the COOH group in an equatorial position have the larger values of the *A* rotational constant (around 3800–3900 MHz). The conformers having the COOH group in an axial position have lower values for the *A* rotational constants (around 2800–3200 MHz). A third family with a *trans*-COOH group in axial arrangement, aligned with the S atom has intermediate values of *A* (3500–3600 MHz). From the values of the rotational constant *A*, the observed rotamers belong to the first two families. Rotamer *a* has a value of *A* = 3215.82492(41) MHz (see Table 1) being identifiable with a form with the COOH group in axial arrangement, while rotamer *b* with a value of *A* = 3803.26722(85) MHz can be identified with a conformer with the COOH group in an equatorial position. The observed rotamers are expected to be the most stable forms of SPro. The predicted most stable forms are I-ax-ce, the global minimum, II-ax-ta, and III-ax-ce at 118 cm^−1^ and 330 cm^−1^ from the global minimum, respectively, according to MP2/6-311++G(2d,p) calculations. B3LYP-D3/6-311++G(2d,p) calculations agree with this energy order and predict other forms at higher energies (see Table S1). The identification of rotamer *a* with conformer II-ax-ta, the most stable type II form, is straightforward based on the reasonable agreement between the predicted and observed rotational parameters (see Table 1 and Table S1). This identification is further confirmed by the observation of intense *μ*_a_-, *μ*_b_-, and *μ*_c_-type spectra which is consistent with the prediction of reasonable high values of the three electric dipole moment components (see Table 1).

If we consider the rotational constants, rotamer *b* could be identified with the global minimum I-ax-ce or with the third form in order of energy, III-ax-ce (see Table 1 and Table S2). Although the rotational constants seem to be non-determinant to distinguish both conformers, the quadrupole coupling constants, highly sensitive to small changes in the orientation of the N atom environment relative to the inertial axis system, indicate that the observed form is the global minimum, conformer I-ax-ce. This assignment is confirmed by the line intensities. In Figure 2, it is possible to observe the 2_0,2_ ← 1_0,1_ transitions for the rotamers assigned. The S/N ratio is bigger for rotamer *a* assigned to conformer II-ax-ta, higher in energy, but with a value of the *μ*_a_ dipole moment higher than that of rotamer *b*, assigned to the global minimum conformer I-ax-ce. Taking into account that conformer III-ax-ce has a lower value of *μ*_a_ dipole moment and is more energetic, the intensity of their *μ*_a_ type lines is expected to be beyond the limit of detection. Both facts confirm the assignation of rotamer *b* to the I-ax-ce predicted conformer of SPro. This identification is in good agreement with the predicted order of energies for both forms and the observation of *μ*_a_-, *μ*_b_-, and *μ*_c_-type spectra according to the electric dipole moment predictions. However, why does rotamer *b,* which corresponds to the global minimum, have a weaker spectrum than that of rotamer *a*, identified with a conformer higher in energy? The answer to this question is on the values of the electric dipole moment components which for the global minimum (*μ*_a_ = 1.0 D, *μ*_b_ = 0.7 D, *μ*_c_ = 0.6 D) are predicted to be smaller than for form II-ax-ta (*μ*_a_ = 2.3 D, *μ*_b_ = 2.6 D, *μ*_c_ = 2.9 D). The observed CP-FTMW spectral intensities depend on the square of the corresponding dipole moment component. Using the predicted electric dipole moment components and the measurements on relative intensities over a series of R-branch *μ*_a_- and *μ*_b_-type lines, an estimation of the relative populations in the jet N*_a_*/N*_b_* = 0.18 (4) is obtained. It has been shown from the rotational spectrum of laser-ablated alanine [44] that the jet conformer relative populations can be related to those calculated at equilibrium at the temperature of the expanding gas. The relative equilibrium populations calculated at room temperature (298 K) from the predicted Gibbs energies (Table 1 and Table S2) for the lowest energy conformers is N_II-ax-ta_/N_I-ax-ce_ = 0.21 in reasonable agreement with the observed relative intensities. 

Two reasons may exist for the non-observation of form III-ax-ce. One is the possible jet relaxation of this conformer to the global minimum I-ax-ce in the supersonic jet. This relaxation occurs through collisions with the carrier gas in the first stages of the expansion when there is an intramolecular vibration connecting both forms by a potential energy barrier below 400 cm^−1^ [45] or even higher (1000 cm^−1^) when there are several degrees of freedom [46,47]. Relaxation from type III to type I forms commonly occurs in α-amino acids without polar side chains in which type III conformers relax to type I forms through the rotation of the COOH group [37,44]. Type III forms have been only observed in molecules as serine [48] or cysteine [49] bearing polar side chains. The MP2/6-311++G(2d,p) potential energy function for the rotation of the COOH group connecting I-ax-ce and III-ax-ce in SPro is shown in Figure S3. The calculated barriers are higher than 600 cm^−1^, but in this case, other large amplitude vibrations such as ring-puckering vibrations may contribute to this relaxation. A second cause for the non-observation of conformer III-ax-ce is the fact that it has a predicted low population and low values of the *μ*_a_ and *μ*_b_ electric dipole moment components. Unfortunately, the predicted *μ*_c_-type spectrum is very weak in the explored frequency region.

### 2.4. Intramolecular Interactions

**Figure 3:**
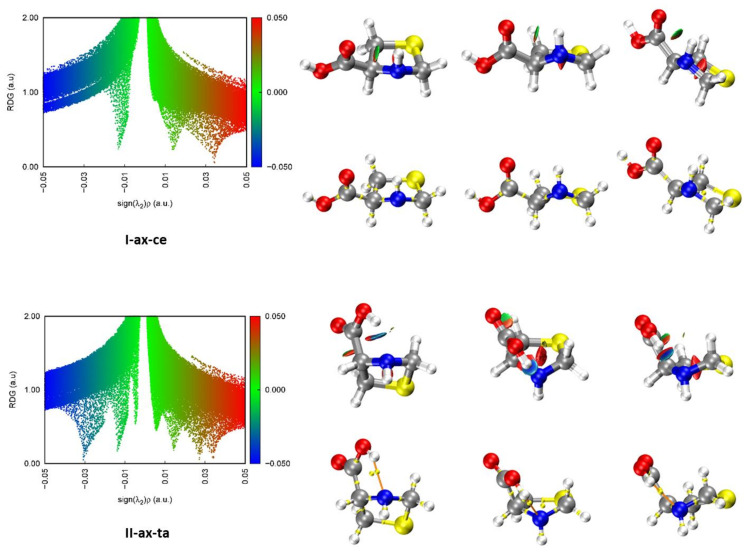
Results of the NCI and QTAIM analyses for the two observed conformers of SPro in the gas phase. Three different perspectives are shown for each conformer. Each point in the scatter graphs on the left side of the figure, corresponds to a grid point in 3D space and represents the reduced density gradient (RDG) vs. sign[*λ*_2_(**r**)]*ρ*(**r**). The spikes on the blue (negative) side correspond to attractive interactions. Those on the red (positive) side correspond to repulsive interactions. The points corresponding to low RDG values (green) represent weak interactions. Those with RGD < 0.5 a.u. are represented in the isosurfaces. Bond critical points (yellow) and bond paths (orange) are also shown.

Aiming to have a better understanding of the nature of the possible hydrogen bond intramolecular interactions stabilizing the conformers of SPro, we have performed quantum theory of atoms in molecules (QTAIM) [50,51] analysis for the different conformers predicted. The molecular graphs corresponding to the observed forms are given in Figure 3. Those for the rest of the conformers are gathered in Figure S4. As can be seen from these Figures, the QTAIM analysis of all forms corresponding to type I or type III arrangements do not show any bond critical point (BCP) revealing the formation of N–H···O=C or N–H···O–H hydrogen bonds, respectively. On the contrary, type II conformers show all of them BCPs and bond paths (BP) evidence of the formation of O–H···N intramolecular hydrogen bonds. BCPs and BPs reveal also an O–H···S hydrogen bond in the form IIS-ax-ta and C_δ_–H···O=C weak hydrogen bond in Ib-ax-ta, ISb-ax-ta and IIISb-ax-ta forms. 

Non-covalent interaction (NCI) analyses [51,52] were also carried out for all the predicted conformers. The results for the observed conformers are illustrated in Figure 3 and those for the rest of the conformers in Figure S5. In the scatter graphs, the *y* and *x*-axis represent respectively the reduced density gradient (RDG) and the sign[*λ*_2_(**r**)]*ρ*(**r**) function. Each point in this graph corresponds to a point in 3D space. *λ*_2_(**r**) is the largest second eigenvalue of the Hessian matrix of the electron density *ρ*(**r**). The strength of weak interactions has a positive correlation with the electron density in the related region; van der Waals interaction regions always have very small values of *ρ*, while the regions corresponding to strong steric effects and hydrogen bonding always have relatively large values of *ρ*. Positive signs indicate repulsive interactions with a depletion of the electron density. A negative sign of *λ*_2_(**r**) means attractive interactions where the electron density is aggregated as in (3, −1) bond critical points. Thus, the product of the sign of *λ*_2_(**r**) and *ρ*(**r**) allows visualizing the non-covalent interactions. The spikes in the low part of the scatter graphs represent the non-covalent interactions present. Those on the left part of the graph represent attractive interactions and those on the right part correspond to repulsive interactions. The isosurfaces give the points with RDG > 0.5. The attractive or repulsive character and the strength of the interactions are shown in the isosurface representation using a color code. As can be seen in the NCI Figure 3 and Figures S4 and S5 for type I or type III forms, which did not show any BCP corresponding to hydrogen bonding, the existence of a weak attractive region between the N–H and C=O or O–H moieties is shown. The existence of attractive stronger interaction regions between the carboxyl O–H and the N atom lone pair confirms the existence of O–H···N hydrogen bonds in type II conformers.

### 2.5. Comparison with Related Systems

**Figure 4:**
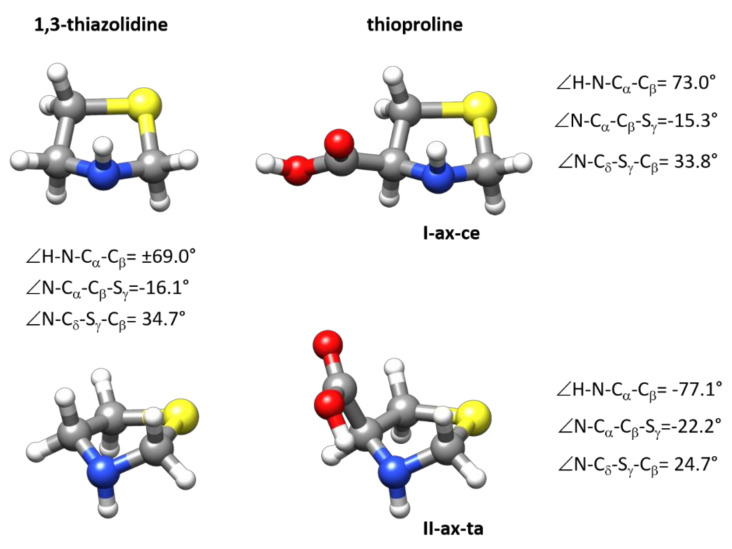
The structures of the two observed forms I-ax-ce and II-ax-ta of SPro are compared to the structure of 1,3-thiazolidine. As can be deduced from the figure these two forms can be interconverted via a concerted movement involving ring puckering and N–H inversion also involved in the inversion of the structure of 1,3-thiazolidine. The figure gives the values of some dihedral angles calculated at MP2/6-311++G(2d,p) level reflecting ring configuration.

The predicted structure of the observed forms is given in the Supplementary Materials (see Table S3). Given the good agreement between the observed and calculated constants (see Table 1), the predicted structures can be taken as reasonable descriptions of the structures of I-ax-ce and II-ax-ta SPro conformers. Both forms have the same configuration of the thiazolidine ring with an axial N-H disposition. In Figure 4, the MP2/6-311++G(2d,p) structures are compared to that of the 1,3-thiazolidine ring calculated at the same level. While the most stable I-ax-ce conformer has ring dihedral angles close to those of 1,3-thiazolidine, the values of these angles for conformer II-ax-ta evidence the distortion of the five-membered ring. In the most stable form, the COOH group adopts an equatorial structure that minimizes interactions. In II-ax-ta form, the O–H···N hydrogen bond interaction stabilization compensates the increase in energy caused by ring-puckering distortions. As shown in Figure 4, both SPro forms can be interconverted via a concerted motion involving ring-puckering vibrations and N–H inversion, the same concerted motion that inverts the structure of 1,3-thiazolidine, together with a rotation of the COOH group which else passes from the *cis*-COOH configuration (OH···O=C intramolecular group interaction) to the *trans*-COOH configuration (HOC=O without intramolecular group interaction) disposition.

The two forms observed for isolated SPro are comparable to those observed for the reference α-amino acid Pro for which four conformers were detected [31,32]. The Pro global minimum and the next conformer in order of energy have the same type II arrangement of the acid and amino groups as in form II-ax-ta, that is, the acid group shows a *trans*-COOH configuration and forms an O–H···N hydrogen bond to the nitrogen atom lone pair. The only difference between these two Pro conformers lies in the pyrrolidine configuration which adopts a C_γ_-*endo* bent configuration for the global minimum and a C_γ_-*exo* bent configuration for the second conformer in order of energy. According to our scheme, the Pro global minimum would be labeled I-ax-ta-C_γ_-*endo* and the second form II-ax-ta-C_γ_-*exo*, so they have the same spatial arrangement of the amino and acid groups in the second most stable form of SPro. The second pair of observed conformers have higher energies (745 and 780 cm^−1^ predicted at MP2/6-311++G(d,p) level) relative to the global minimum. These forms have a type I arrangement of the amino and acid groups similar to that of the I-ax-ce form of SPro and correspond to the two possible C_γ_-*endo* and C_γ_-*exo* configurations of the pyrrolidine ring. These would be labeled as I-ax-te-C_γ_-*endo* and I-ax-te-C_γ_-*exo* and differ from the most stable form of SPro, I-ax-ce, in the orientation of the COOH group. In Pro, as occur in SPro, type III forms were not observed. The substitution of the C_γ_ carbon of Pro by an S atom that forms a thiazolidine ring in SPro has two important consequences in the structure of the α-amino acid. First, it changes the conformational properties reducing the number of configurations of the ring, and second, it reverts the relative energies of type I/type II conformers. In Pro, the global minimum is a type II form while the more stable type I conformers are at moderately high energies. In Spro, the global minimum has a type I arrangement but the next stable type II form is predicted to be not very far from it in energy. Similar situations were found when comparing serine [48] and cysteine [49].

**Figure 5:**
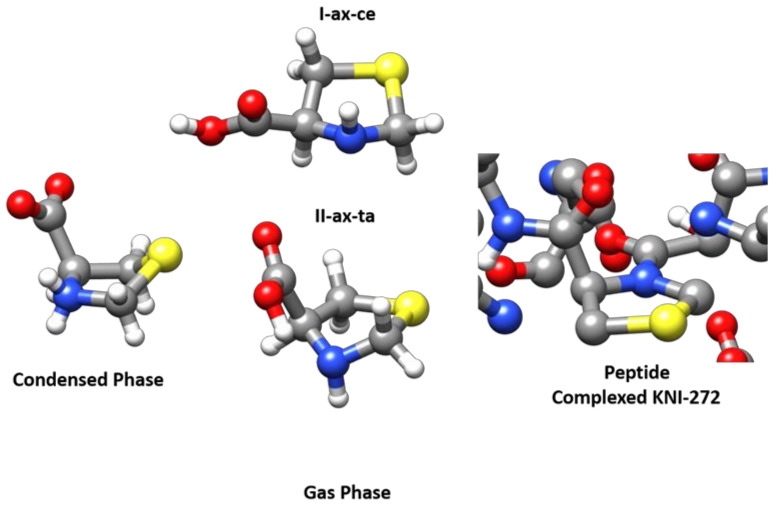
The MP2/6-311++G(2d,p) structures of the two observed forms I-ax-ce and II-ax-ta of SPro are compared to that of the single conformer observed from X-ray diffraction for the zwitterionic form [30,54] (Condensed Phase) in the solid state and with the structure of the SPro fragment observed for KNI complexed to the HIV protease (Peptide complexed KNI-272) [28,55].

To study the possible conformations in solution, we examined the ^1^H-NMR and ^13^C-NMR spectra for 0.1 M solutions performed in D_2_O and DMSO-*d*_6_ (see Figure S7). The ^1^H-NMR spectra of thioproline obtained at pH 7.0 show only one conformation in both ^1^H-NMR and ^13^C-NMR. Only a vicinal coupling constant is observed in the spin system, the dihedral angles corresponding to the observed conformation have not been calculated. In a previous paper [53], the authors show that at pH 6.1, the thiazolidine ring fluctuates rapidly between two extreme and equally probable conformations. Only one conformer of zwitterionic SPro was observed in the solid state by using X-ray diffraction [30]. The solid-state structure retrieved from the Cambridge Structural Database (DSD) [54] can be seen in Figure 5 where the zwitterionic structure is compared with that of the observed neutral forms observed in the gas phase. The ring conformation of the zwitterionic form corresponds approximately to an S_γ_-*endo* bent configuration while in neutral forms the bent atom is nitrogen. This change in the ring conformation is induced by the transformation of cyclic secondary ammonium to a cyclic secondary amine. When SPro forms part of a peptide, it further affects the conformation of the ring since the peptide bond formation implies the transformation of an amine with a pyramidal structure to an amide which tends to be planar as can be observed in Figure 5, where the structure of KNI-272 [28] (RSCB Protein Data Bank [55]) observed when complexed to HIV protease is shown. Thus, the conformation of the SPro thiazolidine ring depends on the functional group associated with the endocyclic nitrogen atom, which can be an ammonium group in the zwitterionic form, and amine in isolated SPro, or an amide when the amino acid forms part of a peptide. 

## 3. Methods

### 3.1. Experimental

A commercial sample of SPro (m.p. 200 °C) was used without further purification. The rotational spectrum was recorded in the 2–8 GHz using a chirped-pulse Fourier transform microwave spectrometer (CP-FTMW) [56] which follows Pate’s design. A new laser ablation source has been incorporated into this instrument [38]. The laser ablation nozzle consists of a standard solenoid valve with the nozzle adapted to a homemade extension cap as described elsewhere [38]. A solid rod of the sample was prepared by grinding the sample and mixing it with a small amount of Cu powder [57] which is pressed to form cylindrical rods of about 1 cm in diameter. The sample rod is held in a channel of appropriate dimensions perpendicular to a small cylinder tube coaxial with the nozzle through which the carrier gas expands (Ar at backing pressures of 2 bar). A small orifice intersecting the gas and sample channels allows the surface of the sample rod to be exposed to the focused laser pulses while the gas expansion drags the laser-vaporized molecules into the supersonic jet. The focused laser beam enters into the nozzle through a channel mutually perpendicular to the gas and sample channels, so that the laser beam crosses the gas channel and hits the sample. The sample rod is continuously rotated and translated back and forth by a stepper motor to ensure that the focused laser pulses hit at a fresh sample surface during all the acquisition time, minimizing the problem of shot-to-shot fluctuation and allowing maximum exploitation of the rod sample. In this work, we used the second (λ = 532 nm) and third harmonic (λ = 355 nm) of an Nd:YAG pulsed laser (Quantel Q-smart 850, 5.2 ns pulse width) with pulse energies of about 20 mJ/pulse. 

The CP-FTMW spectrometer was operated at a repetition rate of 5 Hz following the operation sequence previously described [38] and using gas pulses of 700 μs. Delays between the gas pulse trigger and the laser shot were optimized to have a maximum signal. The vaporized sample is dragged by the gas flow which expands into the vacuum chamber. A chirped pulse of 5 μs exploring a 2 GHz region between 2 and 8 GHz is created by an arbitrary waveform generator and amplified to 20 W. This polarization radiation is emitted by a horn antenna in a direction perpendicular to that of the expanding gas. Once the excitation pulse finishes, the molecular transient emission signal is detected through a second horn, recorded in an interval of 40 μs with a digital oscilloscope, and Fourier-transformed to the frequency domain. The molecular polarization and detection processes are repeated up to eight times per molecular expansion, obtaining up to eight averaged spectra in each molecular jet pulse. This sequence is repeated as soon as the chamber recovers the vacuum conditions to form a new optimum supersonic expansion. The accuracy of the frequency measurements is better than 10 kHz.

The ^1^H-NMR (400 MHz) and ^13^C-NMR (101 MHz) spectra were registered with an Agilent Technologies spectrometer, taking as reference the residual signal of the solvent.

### 3.2. Theoretical

The possible conformers adopted by SPro in the gas phase have been investigated by using CREST [40] and following chemical intuition based on the configuration of thiazolidine [39] and the possible interactions between the carboxylic and amino groups. Structure optimizations of the initial forms were then carried out using density functional theory performed using B3LYP hybrid functional [58] with the 6-311++G(2d,p) [59] basis set and including GD3 [60] empirical dispersion corrections. The geometries of most stable conformers were also optimized at MP2/6-311++G(2d,p) level [61]. Vibrational frequencies were also calculated for the low energy forms at this level from a harmonic force field. These calculations, performed with the G16 package [62], provided the energies and the parameters needed to predict the rotational spectra, that is, the rotational and quadrupole coupling constants, the centrifugal distortion constants, and the components of the electric dipole moment along the inertial axes. To obtain more insight into the nature of the intramolecular interactions responsible for the stabilization of the different forms we have studied the electron density employing the quantum theory of “atoms in molecules” (QTAIM) [50] and noncovalent interaction (NCI) [52] analyses using Multiwfn program [51] using the B3LYP-D3/6-311++G(2d,p) results.

## 4. Conclusions

In this study, we have observed for the first time the rotational spectrum of laser-ablated SPro. Two forms of the α-amino acid have been characterized and identified with the help of DFT and ab initio calculations. Both conformers have a ring configuration close to that of the related compound thiazolidine. The ring has a mixed bent/twisted form with the N atom bent and the N–H bond having an axial configuration. The most abundant and stable conformer has the COOH group in an axial configuration, being on the same side of the ring as the N–H bond. The arrangement of the C=O group with the N–H bond gives rise to a weak interaction which has been characterized from an NCI analysis. Although this interaction does not correspond to a hydrogen bond, the conformer has been identified as having a type I interaction. The second conformer with a *trans*-COOH group in an axial position is forming an O–H···N hydrogen bond which allows classifying this conformer as a type II form. For the related coded amino acid Pro, two type I and two type II forms have been observed differing in the ring configuration. Interestingly, the stability of the equilibrium type I/type II forms is inverted in SPro relative to Pro. Comparison with the structures of the zwitterionic and peptidic forms show that the structure of the ring is conditioned by the function associated with the N atom.

## Figures and Tables

**Table 1:** Experimental rotational parameters for the observed rotamers of thioproline and its comparison to the predicted MP2/6-311++G(2d,p) values (see Figure 1). The predicted electric dipole moment components and relative Gibbs energies are also given.

	Rotamer a	II-ax-ta	Rotamer b	I-ax-ce	III-ax-ce
Param. ^a^	exp	MP2	exp	MP2	MP2
*A*/MHz	3215.82492(41) ^b^	3076.88	3803.26722(85)	3806.75	3835.54
*B*/MHz	1317.61215(14)	1365.99	1091.61856(34)	1084.75	1071.96
*C*/MHz	1089.68026(14)	1136.06	957.57523(39)	960.86	980.62
*κ*	−0.78	−0.76	−0.91	−0.91	−0.94
*D*_J_/kHz	1.4116(24)	0.828	0.145(12)	0.085	0.080
*D*_JK_/kHz	−9.9744(69)	−5.017	0.151(20)	0.193	0.306
*D*_K_/kHz	23.112(71)	10.195	[0.0] ^c^	0.788	0.509
*d*_1_/kHz	−0.02597(59)	−0.021	−0.0225(18)	−0.020	−0.008
*d*_2_/kHz	−0.00317(22)	−0.001	−0.00230(10)	−0.008	−0.002
3/2(χ_aa_)/MHz	−1.9796(31)	−2.14	3.0136(53)	2.93	2.67
1/4(χ_bb_-χ_cc_)/MHz	−0.59941(88)	−0.61	0.1996(18)	0.17	0.01
χ_aa_/MHz	−1.3193(21)	−1.43	2.0091(35)	1.95	1.78
χ_bb_/MHz	−0.5391(28)	−0.51	−0.6053(54)	−0.63	−0.87
χ_cc_/MHz	1.8585(28)	1.93	−1.4037(54)	−1.32	−0.91
*n*	199		93		
*σ*/kHz	4.2		4.9		
*μ*_a_,*μ*_b_,*μ*_c_/D		2.3, 2.6, 2.9		1.0, 0.7, 0.6	0.3, 0.9, 1.3
ΔE/cm^−^^1^		118.4		0.0	332.5
ΔG/cm^−1^		314.5		0.0	370.3

^a^ *A*, *B* and *C* are the rotational constants. *κ* is the Ray asymmetry parameter *κ* = (2*B-A-C*)/(*A-C*). *D*_J_, *D*_JK_, *D*_K_, *d_1_*, and *d*_2_, are the quartic centrifugal distortion constants. χ_aa_, χ_bb,_ and χ_cc_ are the ^14^N quadrupole coupling constants. *n* is the number of quadrupole coupling components fitted. *σ* is the rms deviation of the fit. *μ*_a_,*μ*_b_ and *μ*_c_ are the predicted electric dipole moment components. ΔE is the relative energy to the most stable conformer. ΔG is the predicted relative Gibbs energy calculated at 298.15 K. ^b^ Standard errors in parenthesis in units of the last digit. ^c^ Values in square brackets were kept fixed in the fit.

## Data Availability

Data is contained within the article or supplementary material.

## Supplementary Materials

Complete reference [62]; Figures S1 and S2 show the different conformers of SPro found by exploring the potential energy surface with quantum chemistry methods. Figure S3 shows the potential energy function for the internal rotation of the COOH group interconverting two low-energy forms. Figures S4 and S5 give the results of NCI and QTAIM analysis on all predicted forms. Figure S6 gives the atom numbering scheme. Figure S7 shows the NMR spectrum of SPro. Tables S1 and S2 list the spectroscopic parameters and energies predicted for the different conformers. Table S3: r_e_ structure. Tables S4 and S5: observed frequencies and residuals.

## Sample Availability

Samples of the compounds are commercially available.

## References

[1] Young T.S., Schultz P.G. (2010). Beyond the canonical 20 amino acids: Expanding the genetic lexicon. J. Biol. Chem..

[2] Salwiczek M., Nyakatura E.K., Gerling U.I.M., Ye S., Koksch B. (2012). Fluorinated amino acids: Compatibility with native protein structures and effects on protein–protein interactions. Chem. Soc. Rev..

[3] Pandey A.K., Naduthambi D., Thomas K.M., Zondlo N.J. (2013). Proline editing: A general and practical approach to the synthesis of functionally and structurally diverse peptides. Analysis of steric versus stereoelectronic effects of 4-substituted prolines on conformation within peptides. J. Am. Chem. Soc..

[4] Milner-White E.J., Bell L.H., Maccallum P.H. (1992). Pyrrolidine ring puckering in cis and trans-proline residues in proteins and polypeptides. Different puckers are favoured in certain situations. J. Mol. Biol..

[5] Kim M.K., Kang Y.K. (1999). Positional preference of proline in α-helices. Protein Sci..

[6] Shoulders M.D., Satyshur K.A., Forest K.T., Raines R.T. (2010). Stereoelectronic and steric effects in side chains preorganize a protein main chain. Proc. Natl. Acad. Sci. USA.

[7] Lesarri A., Cocinero E.J., López J.C., Alonso J.L. (2005). Shape of 4(S)- and 4(R)-hydroxyproline in gas phase. J. Am. Chem. Soc..

[8] Bretscher L.E., Jenkins C.L., Taylor K.M., DeRider M.L., Raines R.T. (2001). Conformational stability of collagen relies on a stereoelectronic effect. J. Am. Chem. Soc..

[9] Jenkins C.L., Raines R.T. (2002). Insights on the conformational stability of collagen. Nat. Prod. Rep..

[10] Rather S., Clarke H.T. (1937). The Action of Formaldehyde upon Cysteine. J. Am. Chem. Soc..

[11] Weber H.U., Fleming J.F., Miquel J. (1982). Thiazolidine-4-carboxylic acid, a physiologic sulfhydryl antioxidant with potential value in geriatric medicine. Arch. Gerontol. Geriatr..

[12] De La Fuente M., Ferrández M.D., Del Rio M., Sol Burgos M., Miquel J. (1998). Enhancement of leukocyte functions in aged mice supplemented with the antioxidant thioproline. Mech. Ageing Dev..

[13] Roberts J.C., Nagasawa H.T., Zera R.T., Fricke R.F., Goon D.J.W. (1987). Prodrugs of L-Cysteine as Protective Agents against Acetaminophen-Induced Hepatotoxicity: 2-(Polyhydroxyalkyl)- and 2-(Polyacetoxyalkyl)thiazolidine-4(R)-carboxylic Acids. J. Med. Chem..

[14] Correa R., Del Río M., De La Fuente M. (1999). Improvement of murine immune functions in vitro by thioproline. Immunopharmacology.

[15] Chao T.F., Leu H.B., Huang C.C., Chen J.W., Chan W.L., Lin S.J., Chen S.A. (2012). Thiazolidinediones can prevent new onset atrial fibrillation in patients with non-insulin dependent diabetes. Int. J. Cardiol..

[16] Miquel J., Ramírez-Boscá A., Ramírez-Bosca J.V., Alperi J.D. (2006). Menopause: A review on the role of oxygen stress and favorable effects of dietary antioxidants. Arch. Gerontol. Geriatr..

[17] Frank N., Tsuda M., Ohgaki H., Frei E., Kato T., Sato S. (1990). Detoxifying potential of thioproline against N-nitroso compounds, N-nitrosodimethylamine and N-nitrosocimetidine. Cancer Lett..

[18] Kurashima Y., Tsuda M., Sugimura T. (1990). Marked Formation of Thiazolidine-4-carboxylic Acid, an Effective Nitrite Trapping Agent in Vivo, on Boiling of Dried Shiitake Mushroom (Lentinus edodes). J. Agric. Food Chem..

[19] Suvachittanont W., Kurashima Y., Esumi H., Tsuda M. (1996). Formation of thiazolidine-4-carboxylic acid (thioproline), an effective nitrite-trapping agent in human body, in Parkia speciosa seeds and other edible leguminous seeds in Thailand. Food Chem..

[20] Liu J., Chan W. (2015). Quantification of thiazolidine-4-carboxylic acid in toxicant-exposed cells by isotope-dilution liquid chromatography-mass spectrometry reveals an intrinsic antagonistic response to oxidative stress-induced toxicity. Chem. Res. Toxicol..

[21] Liu J., Chan K.K.J., Chan W. (2016). Identification of Protein Thiazolidination as a Novel Molecular Signature for Oxidative Stress and Formaldehyde Exposure. Chem. Res. Toxicol..

[22] Ham Y.H., Jason Chan K.K., Chan W. (2020). Thioproline Serves as an Efficient Antioxidant Protecting Human Cells from Oxidative Stress and Improves Cell Viability. Chem. Res. Toxicol..

[23] Liu J., Hao C., Wu L., Chan W., Lam H. (2020). Proteomic analysis of thioproline misincorporation in Escherichia coli. J. Proteom..

[24] Baldwin E.T., Bhat T.N., Gulnik S., Liu B., Topol I.A., Kiso Y., Mimoto T., Mitsuya H., Erickson J.W. (1995). Structure of HIV-1 protease with KNI-272, a tight-binding transition-state analog containing allophenylnorstatine. Structure.

[25] Doi M., Ishida T., Katsuya Y., Sasaki M., Taniguchi T., Hasegawa H., Mimoto T., Kiso Y. (2001). KNI-272, a highly selective and potent peptidic HIV protease inhibitor. Acta Crystallogr. Sect. C Cryst. Struct. Commun..

[26] Murcko M.A., Rao B.G., Gomperts R. (1997). Conformational analysis of HIV-1 protease inhibitors: 2. Thioproline P1? Residue in the potent inhibitor KNI-272. J. Comput. Chem..

[27] David L., Luo R., Head M.S., Gilson M.K. (1999). Computational study of KNI-272, a potent inhibitor of HIV-1 protease: On the mechanism of preorganization. J. Phys. Chem. B.

[28] Adachi M., Ohhara T., Kurihara K., Tamada T., Honjo E., Okazaki N., Arai S., Shoyama Y., Kimura K., Matsumura H. (2009). Structure of HIV-1 protease in complex with potent inhibitor KNI-272 determined by high-resolution X-ray and neutron crystallography. Proc. Natl. Acad. Sci. USA.

[29] Kánai K., Podányi B., Bokotey S., Hajdú F., Hermecz I. (2002). Stereoselective sulfoxide formation from a thioproline derivative. Tetrahedron Asymmetry.

[30] Grant N., Ward M.F., Jaspars M., Harrison W.T.A. (2001). (R)-1,3-Thiazolidin-3-ium-4-carboxylate. Acta Crystallogr. Sect. E Struct. Rep. Online.

[31] Lesarri A., Mata S., Cocinero E.J., Blanco S., López J.C., Alonso J.L. (2002). The structure of neutral proline. Angew. Chem. Int. Ed..

[32] Mata S., Vaquero V., Cabezas C., Pena I., Perez C., Lopez J.C., Alonso J.L. (2009). Observation of two new conformers of neutral proline. Phys. Chem. Chem. Phys..

[33] Grabow J.-U., Caminati W. (2009). Microwave Spectroscopy: Experimental Techniques. Front. Mol. Spectrosc..

[34] Pate B.H. (2011). Taking the Pulse of Molecular Rotational Spectroscopy. Science.

[35] Armstrong D.W., Talebi M., Thakur N., Wahab M.F., Mikhonin A.V., Muckle M.T., Neill J.L. (2020). A Gas Chromatography-Molecular Rotational Resonance Spectroscopy Based System of Singular Specificity. Angew. Chem. Int. Ed..

[36] Murugachandran S.I., Tang J., Peña I., Loru D., Sanz M.E. (2021). New Insights into Secondary Organic Aerosol Formation: Water Binding to Limonene. J. Phys. Chem. Lett..

[37] Alonso J.L., López J.C. (2015). Microwave spectroscopy of biomolecular building blocks. Top. Curr. Chem..

[38] Blanco S., Macario A., López J.C. (2021). The structure of isolated thalidomide as reference for its chirality-dependent biological activity: A laser-ablation rotational study. Phys. Chem. Chem. Phys..

[39] Caminati W., Di Bernardo S. (1989). Microwave spectrum and ring puckering motion in thiazolidine. J. Mol. Spectrosc..

[40] Pracht P., Bohle F., Grimme S. (2020). Automated exploration of the low-energy chemical space with fast quantum chemical methods. Phys. Chem. Chem. Phys..

[41] Pickett H.M. (1991). The fitting and prediction of vibration-rotation spectra with spin interactions. J. Mol. Spectrosc..

[42] Watson J.K.G., Durig J.R. (1977). Aspects of Quartic and Sextic Centrifugal Effects on Rotational Energy Levels. Vibrational Spectra and Structure a Series of Advances.

[43] Gordy W., Cook R.L. (1984). Microwave Molecular Spectra.

[44] Blanco S., Lesarri A., López J.C., Alonso J.L. (2004). The gas-phase structure of alanine. J. Am. Chem. Soc..

[45] Ruoff R.S., Klots T.D., Emilsson T., Gutowsky H.S. (1990). Relaxation of conformers and isomers in seeded supersonic jets of inert gases. J. Chem. Phys..

[46] Florio G.M., Christie R.A., Jordan K.D., Zwier T.S. (2002). Conformational preferences of jet-cooled melatonin: Probing trans- and cis-amide regions of the potential energy surface. J. Am. Chem. Soc..

[47] Godfrey P.D., Brown R.D. (1998). Proportions of species observed in jet spectroscopy-vibrational-energy effects: Histamine tautomers and conformers. J. Am. Chem. Soc..

[48] Blanco S., Sanz M.E., López J.C., Alonso J.L. (2007). Revealing the multiple structures of serine. Proc. Natl. Acad. Sci. USA.

[49] Sanz M.E., Blanco S., Löpez J.C., Alonso J.L. (2008). Rotational probes of six conformers of neutral cysteine. Angew. Chem. Int. Ed..

[50] Bader R.F.W. (1991). A quantum theory of molecular structure and its applications. Chem. Rev..

[51] Lu T., Chen F. (2012). Multiwfn: A multifunctional wavefunction analyzer. J. Comput. Chem..

[52] Johnson E.R., Keinan S., Mori-Sanchez P., Contreras-Garcia J., Cohen A.J., Yang W. (2010). Revealing Noncovalent Interactions. J. Am. Chem. Soc..

[53] Piriou F., Lintner K., Lam-Thanh H., Toma F., Fermandjian S. (1978). Synthesis of 13c-labelled [s]-proline and its conformational analysis by nuclear magnetic resonance. Tetrahedron.

[54] Groom C.R., Bruno I.J., Lightfoot M.P., Ward S.C. (2016). IUCr The Cambridge Structural Database. Acta Crystallogr. Sect. B.

[55] Berman H.M., Westbrook J., Feng Z., Gilliland G., Bhat T.N., Weissig H., Shindyalov I.N., Bourne P.E. (2000). The Protein Data Bank. Nucleic Acids Res..

[56] Pinacho P., Blanco S., López J.C. (2019). The complete conformational panorama of formanilide–water complexes: The role of water as a conformational switch. Phys. Chem. Chem. Phys..

[57] Medcraft C., Gougoula E., Bittner D.M., Mullaney J.C., Blanco S., Tew D.P., Walker N.R., Legon A.C. (2017). Molecular geometries and other properties of H_2_O⋯AgI and H_3_ N⋯AgI as characterised by rotational spectroscopy and ab initio calculations. J. Chem. Phys..

[58] Stephens P.J., Devlin F.J., Chabalowski C.F., Frisch M.J. (1994). Ab Initio calculation of vibrational absorption and circular dichroism spectra using density functional force fields. J. Phys. Chem..

[59] Frisch M.J., Pople J.A., Binkley J.S. (1984). Self-consistent molecular orbital methods 25. Supplementary functions for Gaussian basis sets. J. Chem. Phys..

[60] Grimme S., Antony J., Ehrlich S., Krieg H. (2010). A consistent and accurate ab initio parametrization of density functional dispersion correction (DFT-D) for the 94 elements H-Pu. J. Chem. Phys..

[61] Møller C., Plesset M.S. (1934). Note on an approximation treatment for many-electron systems. Phys. Rev..

[62] Frisch M.J., Trucks G.W., Schlegel H.B., Scuseria G.E., Robb M.A., Cheeseman J.R., Scalmani G., Barone V., Petersson G.A., Nakatsuji H. (2016). Gaussian 16, Revision A.03.

