# Supplementary material for: Decoding the Structure of Non-Proteinogenic Amino Acids: The Rotational Spectrum of Jet-Cooled Laser-Ablated Thioproline
Source: Molecules. 2021 Dec 14;26(24):7585. doi: 10.3390/molecules26247585 (PMC8715752; doi:10.3390/molecules26247585)
Supplement: Supplementary file 1 [file molecules-26-07585-s001.zip › molecules-1489346-supplementary.pdf]

# Decoding the Structure of Non-Proteinogenic Amino Acids: The Rotational Spectrum of Jet-Cooled Laser-Ablated Thioproline

**Complete Reference [62]**

**Figure S1**. Predicted conformers of thiazolidine. Relative energies in $cm^{-1}$ calculated at B3LYP-GD3/6-311++G(2d,p). Illustration of conformer labelling.

**Figure S2**. Predicted conformers of SPro showed in 3 different perspectives. See text for labelling. Relative energies in $cm^{-1}$ calculated at B3LYP-GD3/6-311++G(2d,p). The green highlighting remarks the alignment of the COOH group with the S atom.

**Figure S3.** Predicted MP2/6-311++G(2d,p) potential energy function for the internal rotation of COOH group interconverting III-ax-ce and I-ax-ce conformers.

**Figure S4**. QTAIM analysis results for the predicted conformers of SPro in three different perspectives. Bond critical points (yellow) and bond paths (orange) are also shown in the same perspectives as Figures S1 and S2. The green highlighting remarks the alignment of the COOH group with the S atom.

**Figure S5**. Results of the NCI analysis for the predicted conformers of SPro in three different perspectives. The colour code of the energy surfaces is the same as described in Figure 3. The green highlighting remarks the alignment of the COOH group with the S atom.

**Figure S6**. Atom labelling for the I-ax-ce and II-ax-ta detected conformers of SPro.

**Figure S7**: 500 MHz $^1H$ NMR spectra of thioproline (SPro) obtained in $D_2O$ solution at pH 7.0. The spectrum is formed by an ABX system and an AB system.

**Table S1.** Rotational parameters for the SPro conformers predicted at B3LYP-D3/(6-311++G(2d,p)) level.

**Table S2.** Rotational parameters for the most stable conformers of SPro predicted at MP2/(6-311++G(2d,p)) level.

**Table S3.** $r_e$ geometry for I-ax-ce and II-ax-ta detected conformers of TPro (DFT: B3LYP-D3/(6-311++G(2d,p); MP2: MP2/(6-311++G(2d,p)).

**Table S4.** Observed rotational transitions and residuals (all the values in MHz) for the I-ax-ce SPro conformer in the ground vibrational state.

**Table S5.** Observed rotational transitions and residuals (all the values in MHz) for the II-ax-ta SPro conformer in the ground vibrational state.

**Complete Reference [62]**

Gaussian 16, Revision A.03, Frisch, M. J.; Trucks, M. J.; Schlegel, H. B.; Scuseria, G. E.; Robb, M. A.; Cheeseman, J. R.; Scalmani, G.; Barone, V.; Petersson, G. A.; Nakatsuji, H.; Li, X.; Caricato, M.; Marenich, A. V.; Bloino, J.; Janesko, B. G.; Gomperts, R.; Mennucci, B.; Hratchian, H. P.; Ortiz, J. V.;

Izmaylov, A. F.; Sonnenberg, J. L.; Williams-Young, D.; Ding, F.; Lipparini, F.; Egidi, F.; Goings, J.; Peng, B.; Petrone, A.; Henderson, T.; Ranasinghe, D.; Zakrzewski, V. G.; Gao, J.; Rega, N.; Zheng, G.; Liang, W.; Hada, M.; Ehara, M.; Toyota, K.; Fukuda, R.; Hasegawa, J.; Ishida, M.; Nakajima, T.; Honda, Y.; Kitao, O.; Nakai, H.; Vreven, T.; Throssell, K.; Montgomery Jr., J. A.; Peralta, J. E.; Ogliaro, F.; Bearpark, M. J.; Heyd, J. J.; Brothers, E. N.; Kudin, K. N.; Staroverov, V. N.; Keith, T. A.; Kobayashi, R.; Normand, J.; Raghavachari, K.; Rendell, A. P.; Burant, J. C.; Iyengar, S. S.; Tomasi, J.; Cossi, M.; Millam, J. M.; Klene, M.; Adamo, C.; Cammi, R.; Ochterski, J. W.; Martin, R. L.; Morokuma, K.; Farkas, O.; Foresman, J. B.; Fox, D. J. Gaussian, Inc., Wallingford CT, 2016.

**Figure S1**. Predicted axial and equatorial conformers of thiazolidine. Relative energies in $cm^{-1}$ calculated at B3LYP-GD3/6-311++G(2d,p). Illustration of conformer labelling.

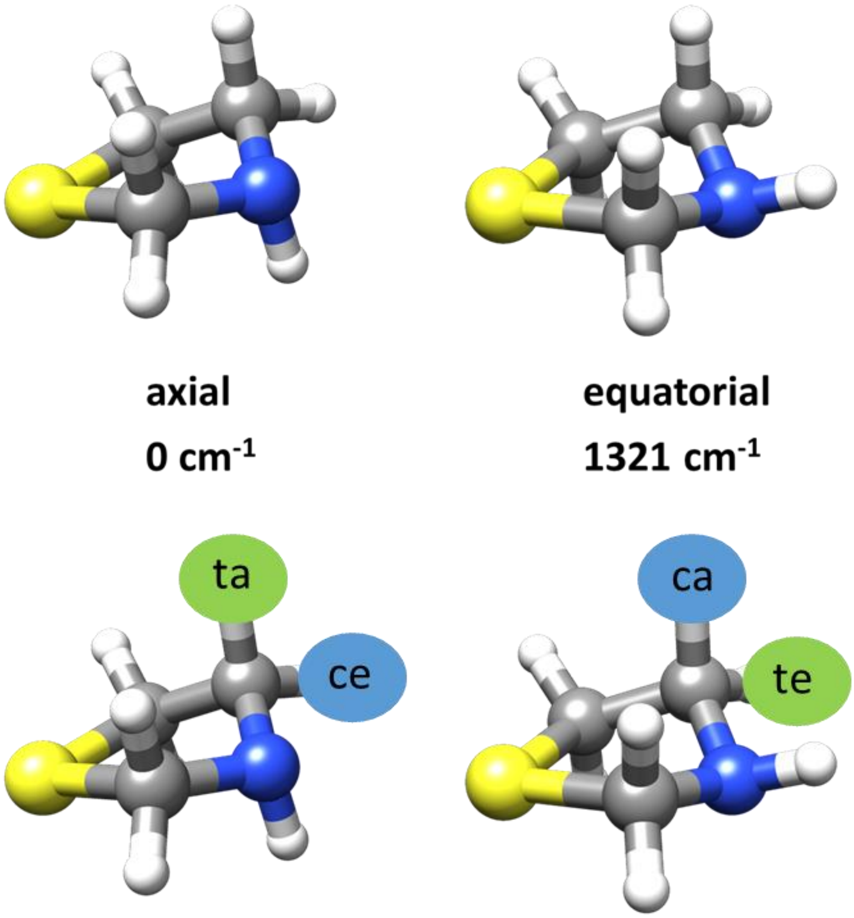


The COOH group will be in the alpha position, that it is remarked with a sphere in the lower part of the figure in order to indicate the possible positions of this group and how it is labelled in the SPro.

"… The third feature is the orientation of the acid group. It can be classified as axial or equatorial but to avoid confusion with the orientation of the N-H group we have chosen to use also the possible arrangements as a function of the *cis* or *trans* orientation of the COOH group relative to the side of the ring of the N-H group. In this way, we can have *trans*-axial (Z=ta), *trans*-equatorial (Z=te), cis-axial (ca), or *cis*-equatorial (Z=ce) orientations of the COOH group."

**Figure S2**. Predicted conformers of SPro showed in 3 different perspectives. See text for labelling. Relative energies in $cm^{-1}$ calculated at B3LYP-GD3/6-311++G(2d,p). The green highlighting remarks the alignment of the COOH group with the S atom.

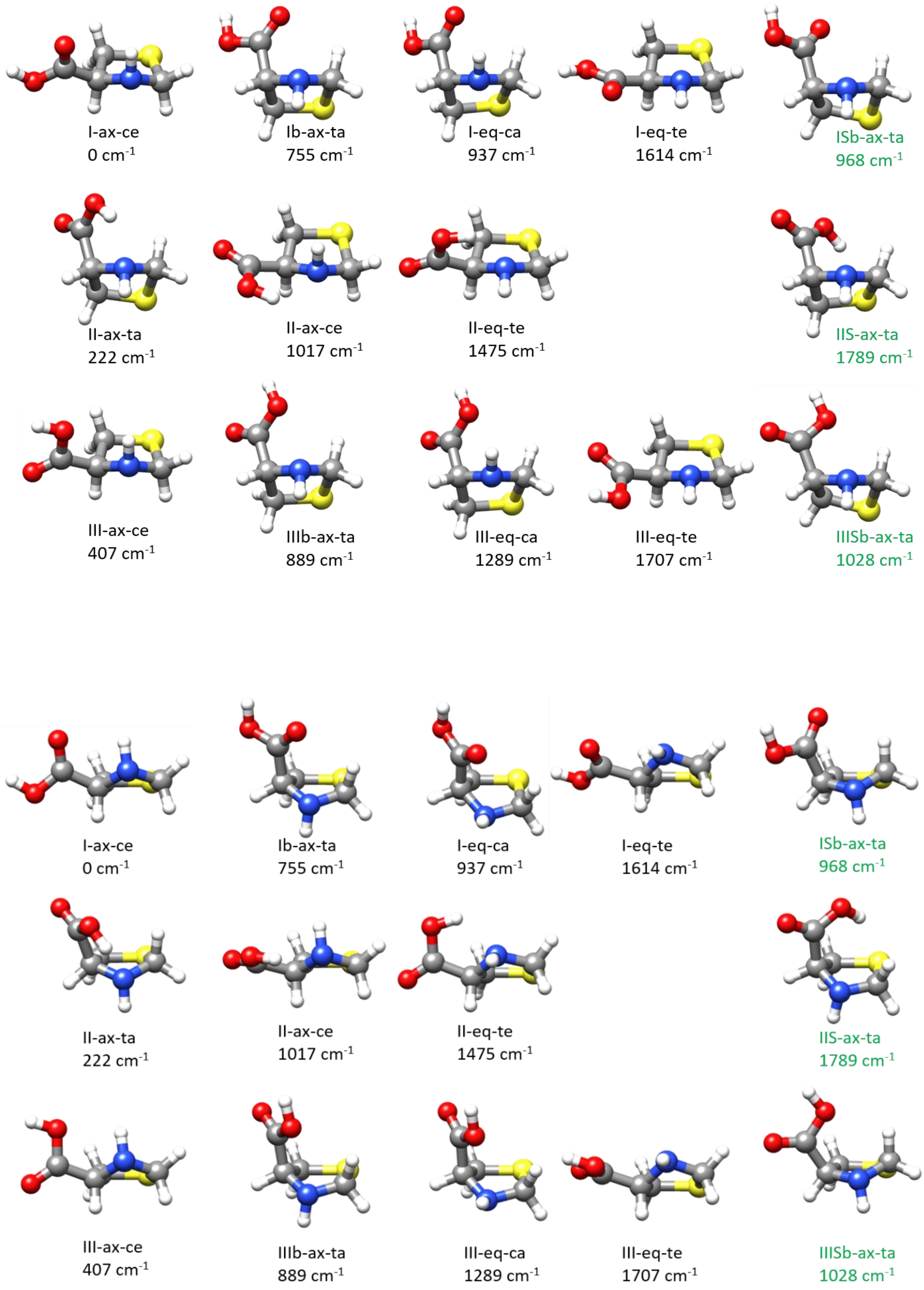

**Figure S2**. Continuation.

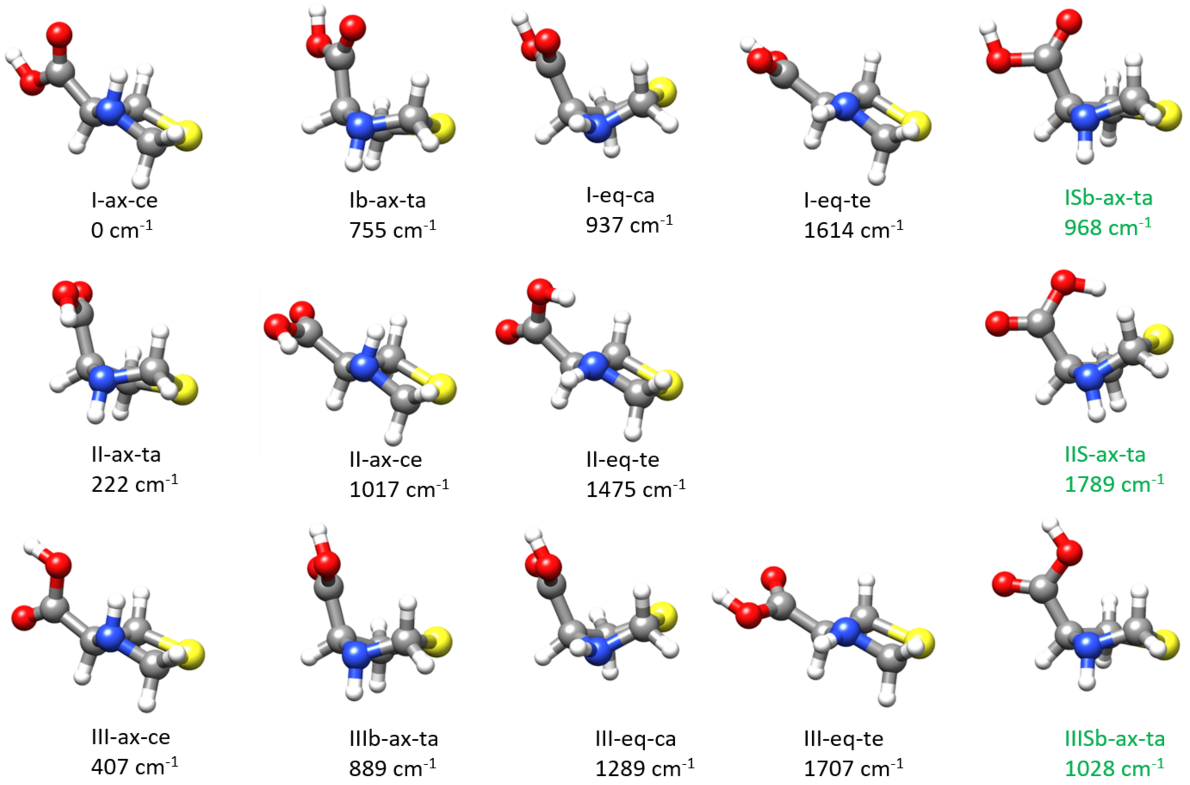

**Figure S3**. Predicted MP2/6-311++G(2d,p) potential energy function for the internal rotation of COOH group interconverting III-ax-ce and I-ax-ce conformers (see Figure S6 for atom labelling).

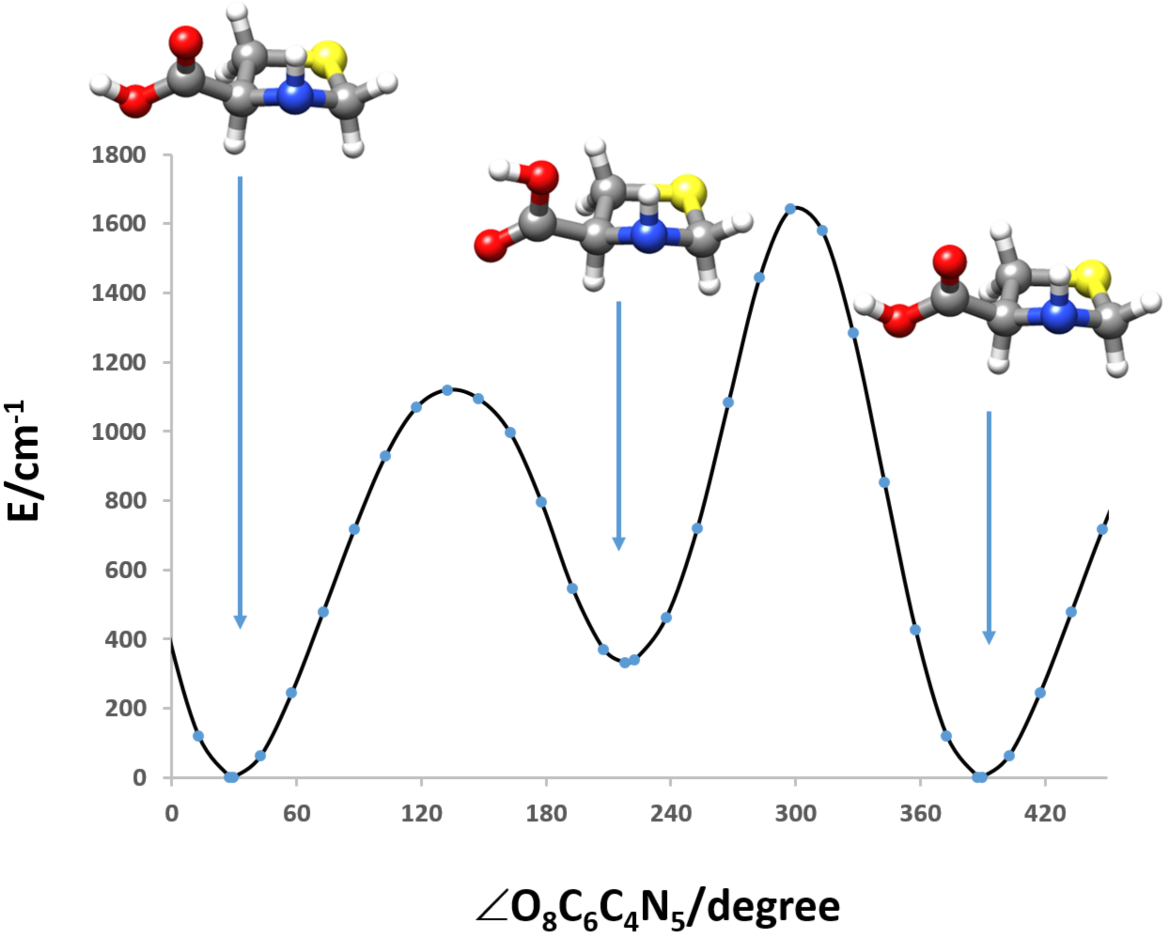

**Figure S4**. QTAIM analysis results for the predicted conformers of SPro in three different perspectives. Bond critical points (yellow) and bond paths (orange) are also shown in the same perspectives as Figures S1 and S2. The green highlighting remarks the alignment of the COOH group with the S atom.

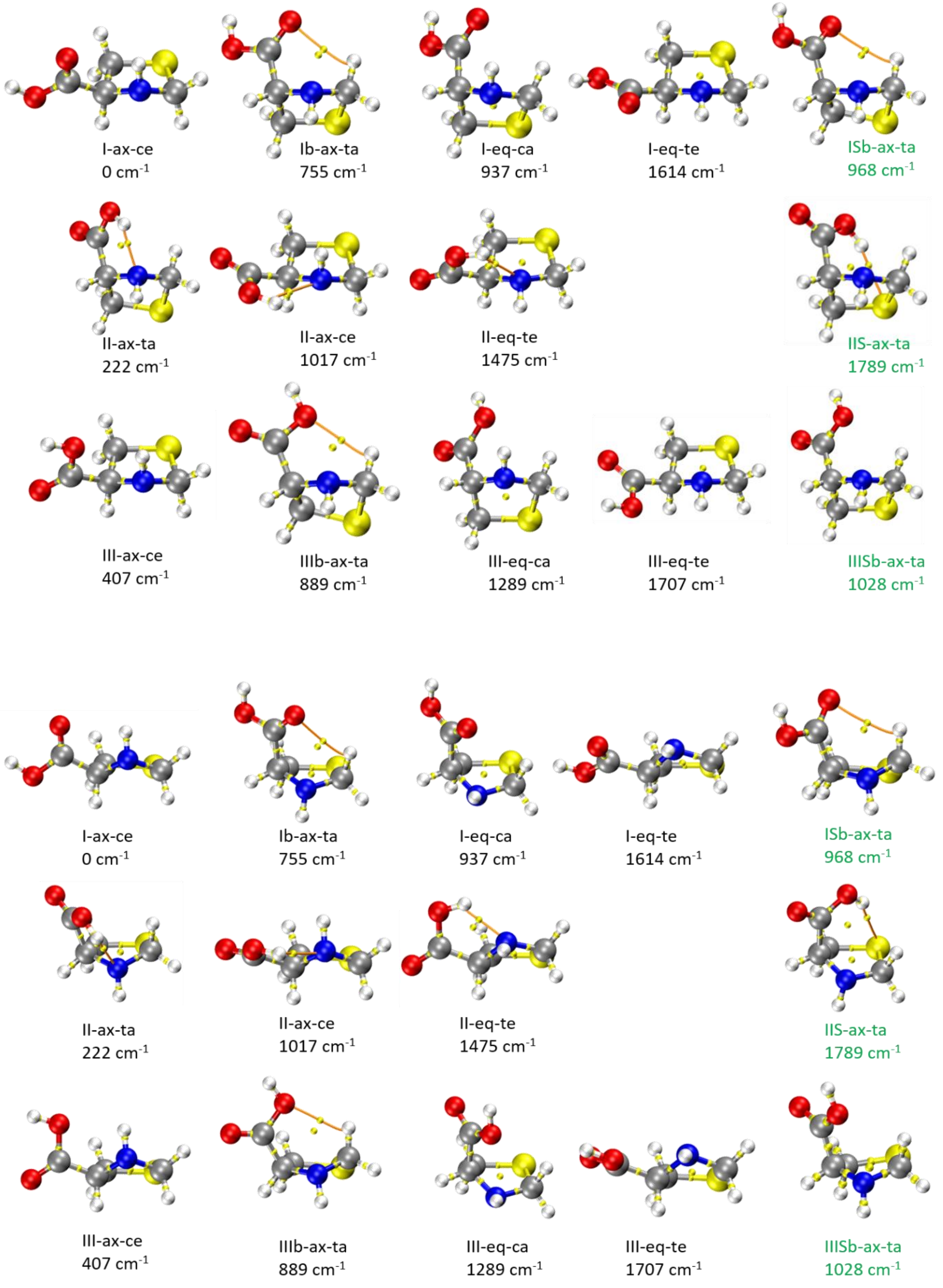

**Figure S4**. Continuation.

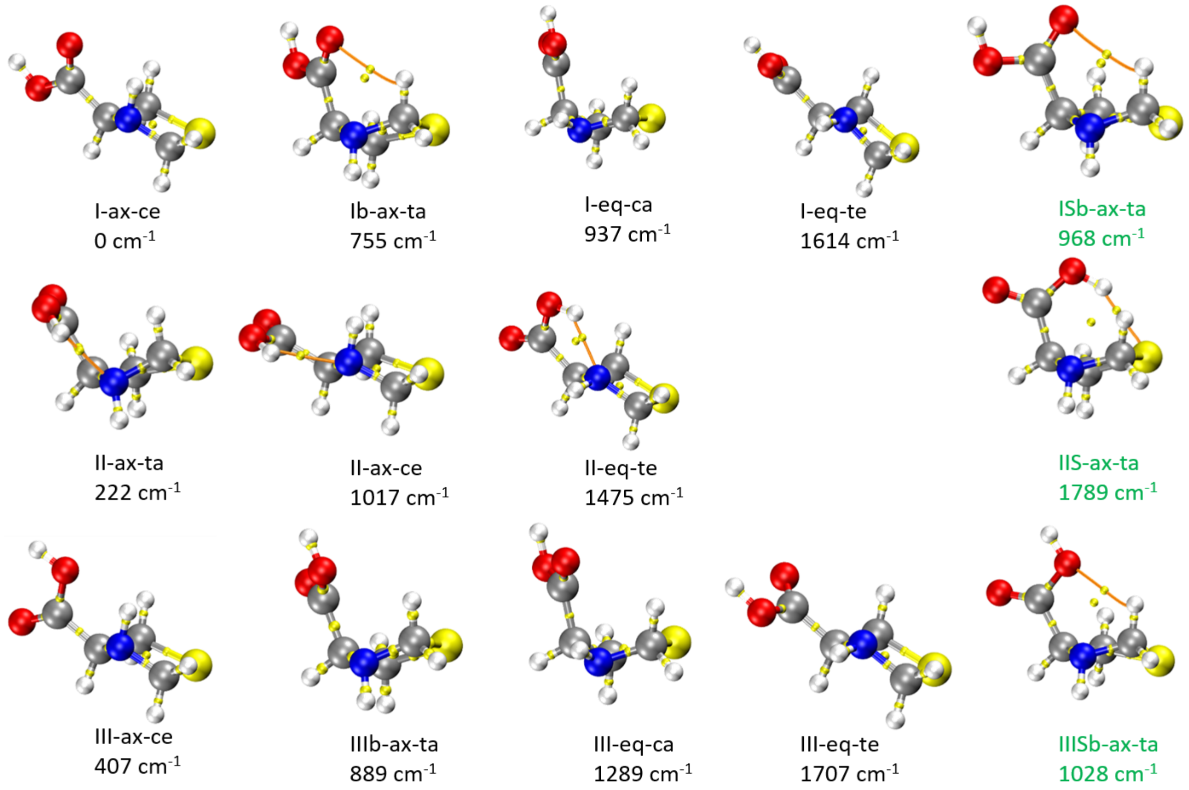

**Figure S5**. Results of the NCI analysis for the predicted conformers of SPro in three different perspectives. The colour code of the energy surfaces is the same as described in Figure 3. The green highlighting remarks the alignment of the COOH group with the S atom.

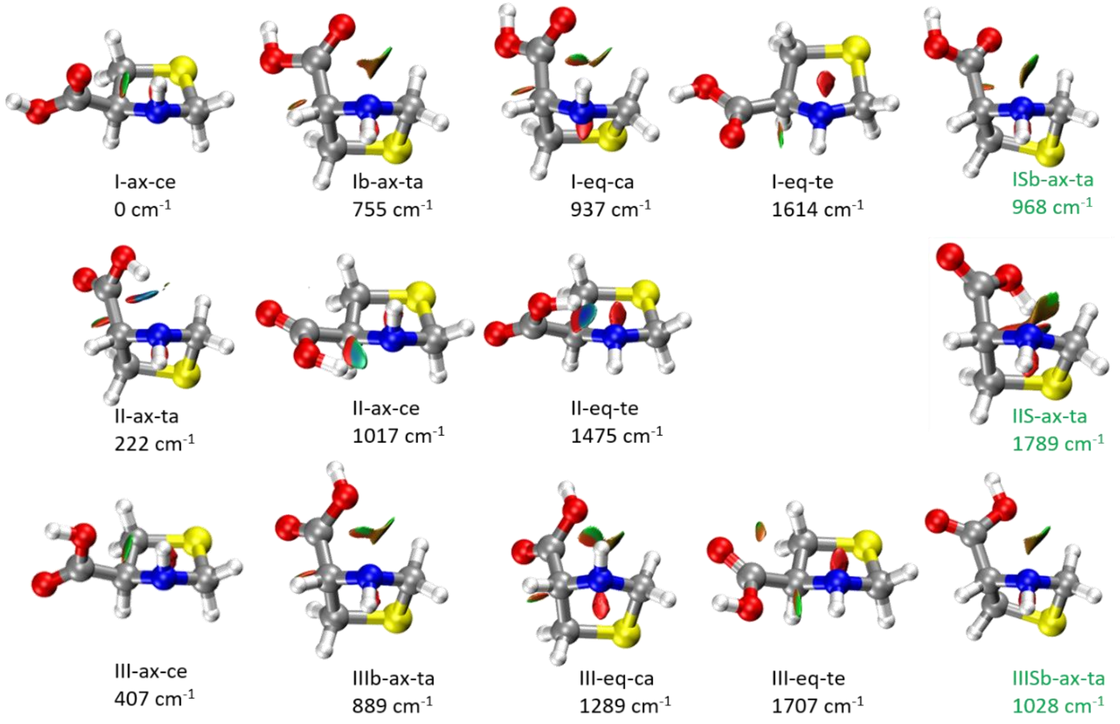


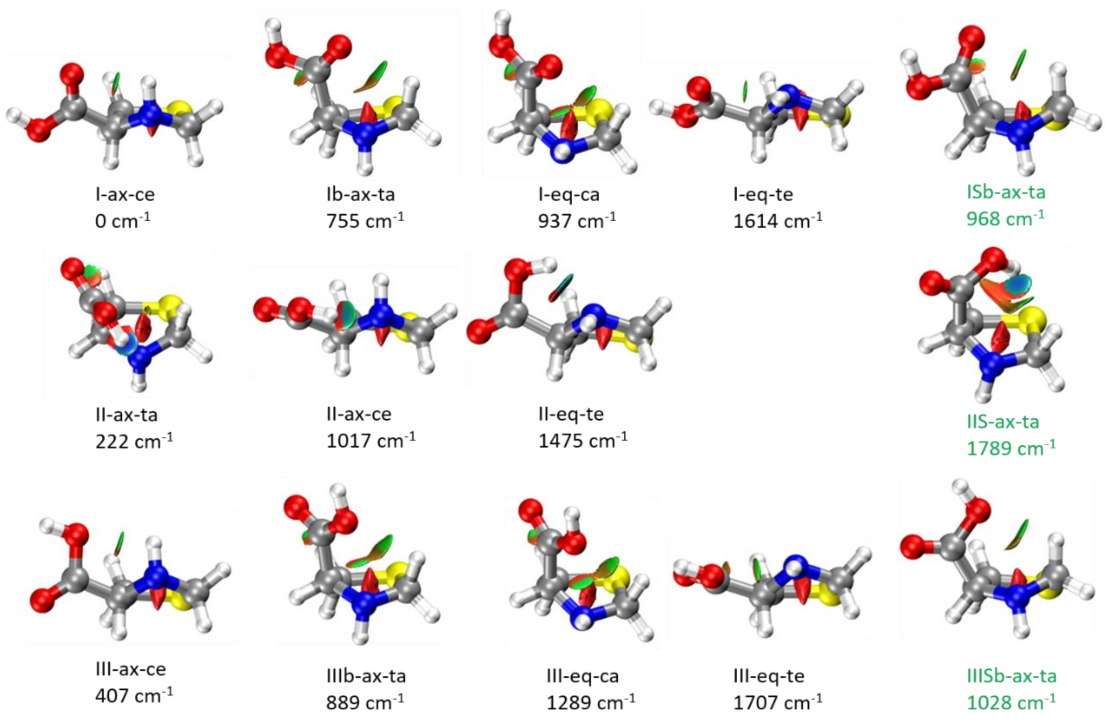

**Figure S5**. Continuation.

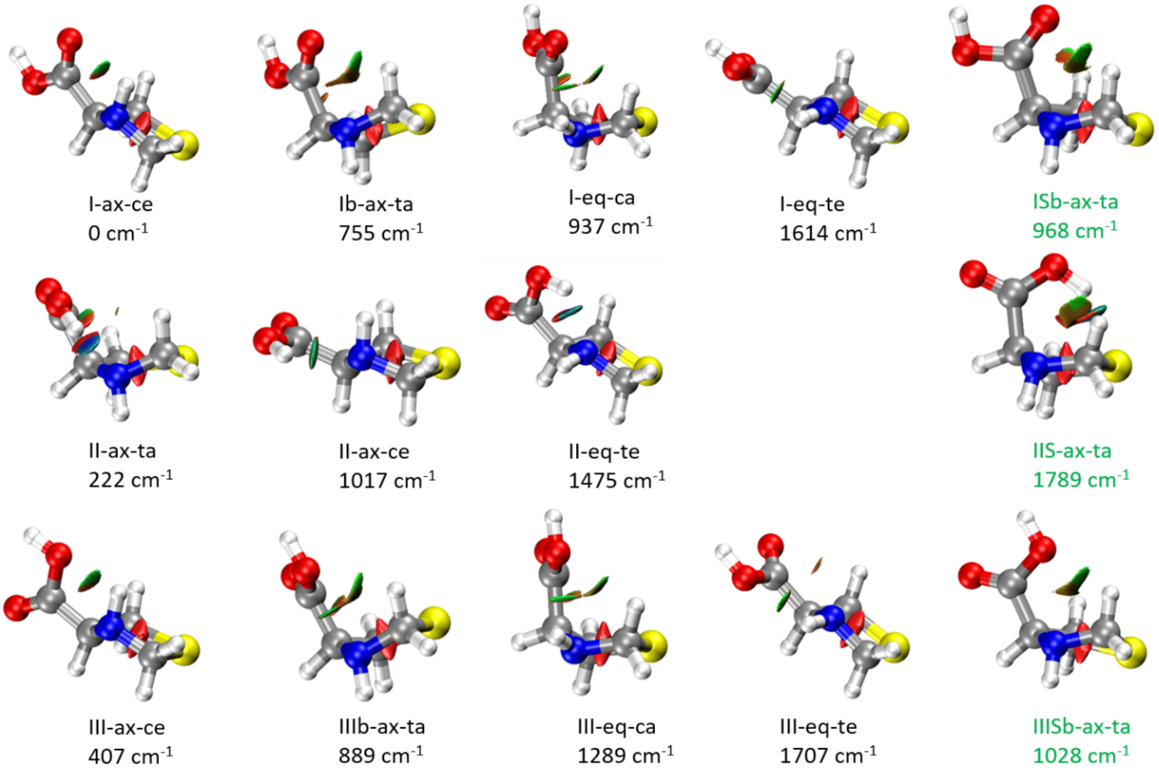

**Figure S6**. Atom labelling for the I-ax-ce and II-ax-ta detected conformers of SPro.

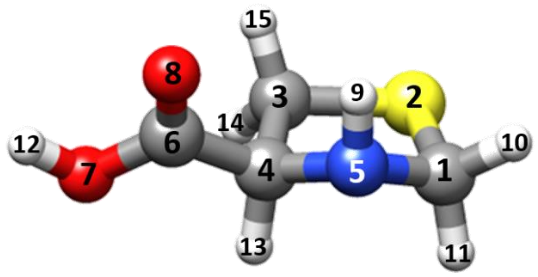


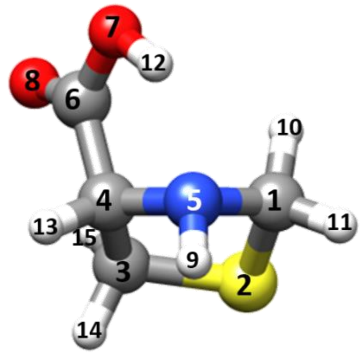

**Figure S7**: 500 MHz $^{1}$H NMR spectra of thioproline(SPro) obtained in $D_2O$ solution at pH 7.0. The spectrum is formed by an ABX system and an AB system.

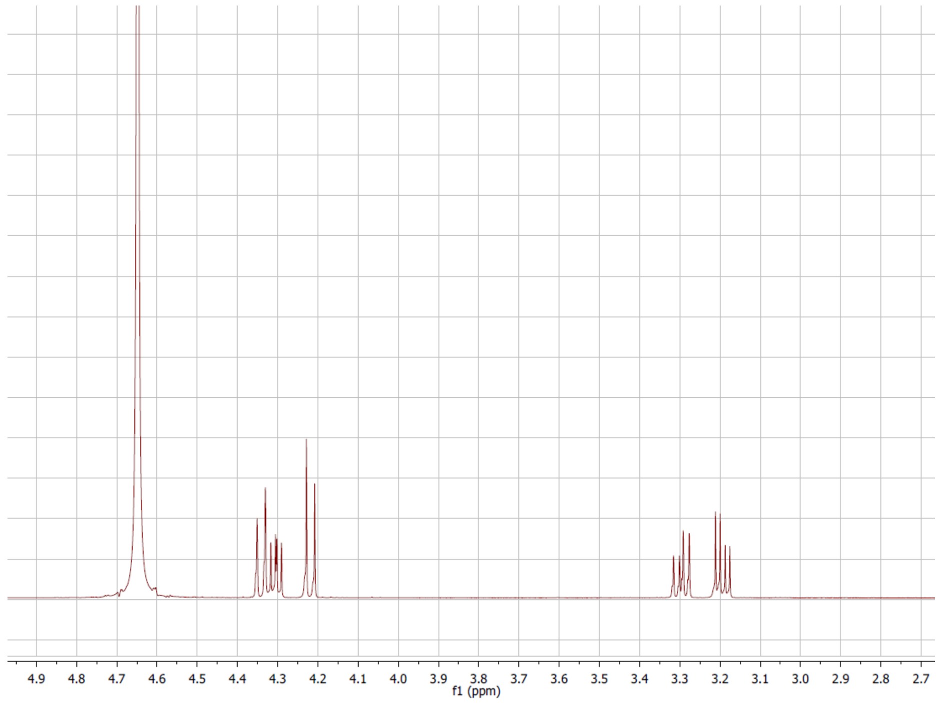


In order to study the possible conformations existing in solution for thioproline (SPro), we examined by $^{1}$H NMR and $^{13}$C NMR in 0,1 M solution in $D_2O$. The $H^{A}_{5}$ , $H^{B}_{5}$ protons form the AB part of an ABX system (X = $H_4$). The coupling constants of $^{3}J$ $H_4$-$H^{A}_{5}$ and $^{3}J$ $H_4$-$H^{B}_{5}$ are parameters that can be utilized in structure determination. They lead to conformationally reasonable values of the dihedral angles in thioproline. The coupling constants of the hydrogens attached to carbons 4 and 5 ($^{3}J$ $H_4$-$H^{A}_{5}$ = 5.8 Hz and $^{3}J$ $H_4$-$H^{B}_{5}$ = 7.4 Hz) allowed us to assume dihedral angles $H_4$-$C_4$-$C_5$-$H^{A}_{5}$ and $H_4$-$C_4$-$C_5$-$H^{B}_{5}$ approximately 30º and 130º, respectively, according to the curve by Kopple [1]. The observed conformation is not planar, the probable conformation in solution correspond to $S_{exo}$ form, relative to the orientation *anti* of the S atom in respect of the carboxyl group.

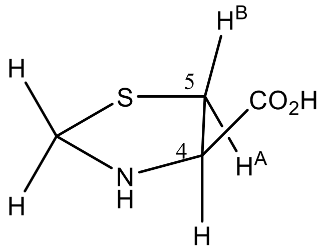


Thioproline (SPro)

1. Kopple, K. D.; Wiley, G. R. and Tauke, R. *Biopolymers* **1973**, 12, 627-636.

**Table S1.** Rotational parameters for the SPro conformers predicted at B3LYP-D3/(6-311++G(2d,p)) level.

| Param.[a] | **I-ax-ce** | **II-ax-ta** | **III-ax-ce** | **Ib-ax-ta** | **III-ax-ta** | **I-eq-ca** | **ISb-ax-ta** |
|---|---|---|---|---|---|---|---|
| $A$/MHz | 3775.74 | 3125.48 | 3798.10 | 2970.97 | 2874.27 | 2850.97 | 3608.40 |
| $B$/MHz | 1079.47 | 1322.18 | 1065.84 | 1339.11 | 1390.92 | 1413.94 | 1157.11 |
| $C$/MHz | 950.35 | 1094.73 | 968.17 | 1180.32 | 1190.65 | 1211.60 | 1111.71 |
| $\kappa$ | -0.91 | -0.78 | -0.93 | -0.82 | -0.76 | -0.75 | -0.96 |
| $P_{aa}$/uÅ$^2$ | 433.05 | 341.09 | 431.55 | 317.73 | 305.98 | 298.64 | 375.65 |
| $P_{bb}$/uÅ$^2$ | 98.73 | 120.56 | 90.45 | 110.44 | 118.47 | 118.48 | 78.95 |
| $P_{cc}$/uÅ$^2$ | 35.12 | 41.14 | 42.61 | 59.67 | 57.36 | 58.79 | 61.11 |
| $3/2(\chi_{aa})$/MHz | 3.16 | -2.19 | 2.88 | -0.09 | -1.04 | 0.02 | -1.93 |
| $1/4(\chi_{bb}-\chi_{cc})$/MHz | 0.22 | -0.65 | 0.07 | -1.18 | -1.04 | 1.29 | -0.25 |
| $\chi_{aa}$/MHz | 2.11 | -1.46 | 1.92 | -0.06 | -0.69 | 0.01 | -1.29 |
| $\chi_{bb}$/MHz | -0.62 | -0.57 | -0.82 | -2.33 | -1.73 | 2.58 | 0.15 |
| $\chi_{cc}$/MHz | -1.49 | 2.03 | -1.11 | 2.39 | 2.42 | -2.59 | 1.13 |
| $\mu_a$/D | -1.06 | 2.38 | -0.33 | -0.67 | 0.06 | -1.50 | 1.25 |
| $\mu_b$/D | -0.69 | 2.73 | 1.12 | -1.11 | 1.62 | -0.48 | 0.89 |
| $\mu_c$/D | 0.76 | 2.82 | -1.34 | 2.09 | 1.45 | 0.94 | 1.92 |
| $\mu_T$/D | 1.48 | 4.59 | 1.78 | 2.46 | 2.18 | 0.94 | 2.46 |
| E/ h | -760.1966725 | -760.1956576 | -760.1948158 | -760.1932292 | -760.1926191 | -760.1923991 | -760.1922594 |
| $\Delta$E /cm$^{-1}$ | 0.0 | 222.74 | 407.50 | 755.72 | 889.62 | 937.92 | 968.56 |
| $\Delta$E / kJ mol$^{-1}$ | 0.0 | 2.66 | 4.87 | 9.04 | 10.64 | 11.22 | 11.59 |

[a] $A$, $B$ and $C$ are the rotational constants. $\kappa$ is the Ray asymmetry parameter $\kappa=(2B-A-C)/(A-C)$. $P_{\alpha\alpha}$ ($\alpha$ = a, b, c) are the planar moments of inertia, derived from the inertial moments $P_{cc}=(I_a+I_b-I_c)/2$. $\chi_{aa}$, $\chi_{bb}$ and $\chi_{cc}$ are the $^{14}$N quadrupole coupling constants. $\mu_a$,$\mu_b$,$\mu_c$ are the electric dipole moment components. $n$ is the number of quadrupole coupling components fitted. E is the electronic energy in Hartree. $\Delta$E is the relative energy to the most stable conformer.

**Table S1.** Continuation

| Param.[a] | **II-ax-ce** | **IIISb-ax-ta** | **III-eq-ca** | **II-eq-te** | **I-eq-te** | **III-eq-te** | **IIS-ax-ta** |
|---|---|---|---|---|---|---|---|
| $A$/MHz | 3810.53 | 3525.85 | 2843.06 | 3799.96 | 3862.29 | 3906.56 | 3085.68 |
| $B$/MHz | 1123.98 | 1168.05 | 1419.97 | 1098.51 | 1116.52 | 1112.45 | 1345.21 |
| $C$/MHz | 897.57 | 1106.51 | 1210.41 | 963.92 | 897.43 | 897.72 | 1242.01 |
| $\kappa$ | -0.84 | -0.95 | -0.74 | -0.91 | -0.85 | -0.86 | -0.89 |
| $P_{aa}$/uÅ$^2$ | 440.03 | 373.03 | 297.84 | 425.68 | 442.47 | 443.94 | 309.41 |
| $P_{bb}$/uÅ$^2$ | 123.02 | 83.70 | 119.69 | 98.62 | 120.68 | 119.01 | 97.50 |
| $P_{cc}$/uÅ$^2$ | 9.60 | 59.64 | 58.07 | 34.38 | 10.17 | 10.35 | 66.28 |
| $3/2(\chi_{aa})$/MHz | 1.65 | -1.09 | -0.39 | 3.99 | 4.77 | 4.55 | -1.23 |
| $1/4(\chi_{bb}-\chi_{cc})$/MHz | -0.78 | -0.92 | 1.28 | 1.68 | 2.11 | 1.87 | 0.44 |
| $\chi_{aa}$/MHz | 1.10 | -0.73 | -0.26 | 2.66 | 3.18 | 3.03 | -0.82 |
| $\chi_{bb}$/MHz | -2.12 | -1.47 | 2.69 | 2.03 | 2.62 | 2.22 | 1.29 |
| $\chi_{cc}$/MHz | 1.02 | 2.19 | -2.43 | -4.69 | -5.80 | -5.25 | -0.47 |
| $\mu_a$/D | 2.21 | 0.55 | -1.15 | 3.12 | -0.92 | -1.15 | -4.30 |
| $\mu_b$/D | 2.72 | 0.62 | 2.22 | 2.47 | -0.37 | 2.45 | -1.61 |
| $\mu_c$/D | 0.23 | 0.04 | 0.46 | 0.90 | 1.38 | 1.23 | 1.63 |
| $\mu_T$/D | 3.52 | 0.83 | 2.55 | 4.08 | 1.70 | 2.97 | 4.87 |
| E/ h | -760.192038 | -760.191988 | -760.1907986 | -760.1899494 | -760.1893161 | -760.1888934 | -760.1885202 |
| ΔE /cm$^{-1}$ | 1017.16 | 1028.15 | 1289.17 | 1475.55 | 1614.54 | 1707.32 | 1789.22 |
| ΔE / kJ mol$^{-1}$ | 12.17 | 12.30 | 15.42 | 17.65 | 19.31 | 20.42 | 21.40 |

**Table S2.** Rotational parameters for the most stable conformers of SPro predicted at MP2/(6-311++G(2d,p)) level.

| Param.[a] | **I-ax-ce** | **II-ax-ta** | **III-ax-ce** |
|---|---|---|---|
| $A$/MHz | 3806.75 | 3076.88 | 3835.54 |
| $B$/MHz | 1084.75 | 1365.99 | 1071.96 |
| $C$/MHz | 960.86 | 1136.06 | 980.62 |
| $\kappa$ | -0.91 | -0.76 | -0.94 |
| $P_{aa}$/uÅ$^2$ | 429.6 | 325.3 | 427.5 |
| $P_{bb}$/uÅ$^2$ | 96.4 | 119.6 | 87.8 |
| $P_{cc}$/uÅ$^2$ | 36.3 | 44.7 | 43.9 |
| $D_J$/kHz | 0.085 | 0.828 | 0.080 |
| $D_{JK}$/kHz | 0.193 | -5.017 | 0.306 |
| $D_K$/kHz | 0.788 | 10.195 | 0.509 |
| $d_1$/kHz | -0.020 | -0.021 | -0.008 |
| $d_2$/kHz | -0.008 | -0.001 | -0.002 |
| $3/2(\chi_{aa})$/MHz | 2.93 | -2.14 | 2.67 |
| $1/4(\chi_{bb}-\chi_{cc})$/MHz | 0.17 | -0.61 | 0.01 |
| $\chi_{aa}$/MHz | 1.95 | -1.43 | 1.78 |
| $\chi_{bb}$/MHz | -0.63 | -0.51 | -0.87 |
| $\chi_{cc}$/MHz | -1.32 | 1.93 | -0.91 |
| $\mu_a$/D | 1.03 | 2.30 | 0.32 |
| $\mu_b$/D | 0.72 | 2.62 | 0.86 |
| $\mu_c$/D | 0.61 | 2.87 | 1.33 |
| $\mu_T$/D | 1.40 | 4.52 | 1.62 |
| E/$E_h$ | -758.7399655 | -758.739426 | -758.7384506 |
| ΔE /cm$^{-1}$ | 0.00 | 118.41 | 332.48 |
| ΔE / kJ mol$^{-1}$ | 0.00 | 1.42 | 3.98 |
| $E_{Gibbs}$ / h | -758.655017 | -758.653584 | -758.65333 |
| Δ$E_{Gibbs}$ /cm$^{-1}$ | 0.00 | 314.51 | 370.25 |
| Δ$E_{Gibbs}$ / kJ mol$^{-1}$ | 0.00 | 3.76 | 4.43 |

[a] $A$, $B$ and $C$ are the rotational constants. $\kappa$ is the Ray asymmetry parameter $\kappa$=(2$B$-$A$-$C$)/($A$-$C$). $P_{\alpha\alpha}$ ($\alpha$ = a, b, c) are the planar moments of inertia, derived from the inertial moments $P_{cc}=(I_a+I_b-I_c)/2$. $D_J$, $D_{JK}$, $D_K$, $d_1$, and $d_2$, are the quartic centrifugal distortion constants. $\chi_{aa}$, $\chi_{bb}$ and $\chi_{cc}$ are the $^{14}$N quadrupole coupling constants. $\mu$ is the dipole moment in Debay. E is the electronic energy in Hatrees, ΔE is the relative energy to the global minimum. $E_{Gibbs}$ is the Gibbs energy in Hatrees calculated at 298.15 K, Δ$E_{Gibbs}$ is the relative Gibbs energy to the global minimum.

**Table S3.** $r_e$ geometry for I-ax-ce and II-ax-ta detected conformers of SPro (DFT: B3LYP-D3/(6-311++G(2d,p); MP2: MP2/(6-311++G(2d,p)

| I-ax-ce | DFT | | | MP2 | | |
|---|---|---|---|---|---|---|
| atom[a] | *a*[b] | *b* | *c* | *a* | *b* | *c* |
| C1 | 1.706346 | 1.135542 | 0.444810 | -1.700381 | 1.117842 | -0.455048 |
| O2 | 2.197678 | -0.562844 | -0.232949 | -2.188466 | -0.558669 | 0.225158 |
| C3 | 0.442070 | -1.104036 | -0.257636 | -0.440188 | -1.085679 | 0.286861 |
| C4 | -0.352574 | 0.019912 | 0.477511 | 0.347204 | 0.012216 | -0.471956 |
| N5 | 0.292984 | 1.300649 | 0.250652 | -0.287036 | 1.297953 | -0.248714 |
| C6 | -1.777125 | 0.047681 | -0.030627 | 1.767423 | 0.044486 | 0.028502 |
| O7 | -2.550116 | -0.857918 | 0.606235 | 2.547551 | -0.843307 | -0.630587 |
| O8 | -2.173811 | 0.745884 | -0.926995 | 2.158289 | 0.732505 | 0.944114 |
| H9 | 0.077231 | 1.621002 | -0.689279 | -0.091232 | 1.590598 | 0.707962 |
| H10 | 2.272657 | 1.908652 | -0.069484 | -2.273886 | 1.895582 | 0.046143 |
| H11 | 1.952608 | 1.142721 | 1.506028 | -1.925420 | 1.121671 | -1.521242 |
| H12 | -3.429429 | -0.828736 | 0.196158 | 3.424686 | -0.802306 | -0.210842 |
| H13 | -0.354722 | -0.197877 | 1.547547 | 0.333977 | -0.214663 | -1.540884 |
| H14 | 0.332666 | -2.061352 | 0.248864 | -0.320464 | -2.059711 | -0.187158 |
| H15 | 0.108703 | -1.202409 | -1.291359 | -0.109141 | -1.141855 | 1.325636 |

| II-ax-ta | DFT | | | MP2 | | |
|---|---|---|---|---|---|---|
| atom[a] | *a*[b] | *b* | *c* | *a* | *b* | *c* |
| C1 | 1.119172 | 1.224949 | -0.445523 | 1.056786 | 1.208612 | -0.483343 |
| O2 | 2.045276 | -0.386547 | -0.366017 | 1.998374 | -0.377676 | -0.386849 |
| C3 | 0.674857 | -1.177396 | 0.582179 | 0.699625 | -1.136189 | 0.661966 |
| C4 | -0.374718 | -0.073196 | 0.853190 | -0.367748 | -0.052649 | 0.886829 |
| N5 | 0.208455 | 1.264908 | 0.687518 | 0.193695 | 1.286889 | 0.685433 |
| C6 | -1.617141 | -0.218457 | -0.050449 | -1.572024 | -0.235280 | -0.049517 |
| O7 | -2.178179 | 0.951678 | -0.393254 | -2.170192 | 0.921763 | -0.390117 |
| O8 | -2.073167 | -1.278331 | -0.377279 | -1.980678 | -1.312906 | -0.404960 |
| H9 | 0.710841 | 1.530347 | 1.527475 | 0.745810 | 1.550271 | 1.498419 |
| H10 | 0.551459 | 1.262714 | -1.375535 | 0.443617 | 1.210100 | -1.385447 |
| H11 | 1.822301 | 2.054425 | -0.417140 | 1.751491 | 2.046424 | -0.513874 |
| H12 | -1.593604 | 1.651090 | -0.033453 | -1.604839 | 1.627631 | -0.006628 |
| H13 | -0.758966 | -0.160508 | 1.872865 | -0.771563 | -0.116086 | 1.902042 |
| H14 | 1.085288 | -1.567098 | 1.511871 | 1.150982 | -1.442068 | 1.605053 |
| H15 | 0.229790 | -1.992625 | 0.016422 | 0.257729 | -2.001688 | 0.170990 |

[a] Atom labelling, see Figure S6. [b] *a*, *b*, and *c* are the coordinates in the principal axis system of the conformer in Å.

**Table S4.** Observed rotational transitions and residuals (all the values in MHz) for the I-ax-ce SPro conformer in the ground vibrational state.

| J′ | $K_{-1}$′ | $K_{+1}$′ | J′′ | $K_{-1}$′′ | $K_{+1}$′′ | F′ | F′′ | Obs. | Obs.-Cal. |
|---|---|---|---|---|---|---|---|---|---|
| 1 | 1 | 1 | 0 | 0 | 0 | 1 | 1 | 4760.6866 | -0.0037 |
| | | | | | | 2 | 1 | 4760.8748 | 0.0029 |
| | | | | | | 0 | 1 | 4761.1295 | -0.0148 |
| 1 | 1 | 0 | 0 | 0 | 0 | 2 | 1 | 4894.9575 | 0.0024 |
| 2 | 0 | 2 | 1 | 0 | 1 | 1 | 1 | 4092.5225 | -0.0040 |
| | | | | | | 3 | 2 | 4093.4930 | 0.0018 |
| | | | | | | 1 | 0 | 4094.0363 | 0.0029 |
| | | | | | | 2 | 2 | 4094.1467 | 0.0041 |
| 2 | 1 | 1 | 1 | 1 | 0 | 3 | 1 | 4232.3078 | -0.0037 |
| | | | | | | 2 | 2 | 4232.5207 | 0.0146 |
| | | | | | | 1 | 0 | 4232.6281 | 0.0032 |
| | | | | | | 2 | 2 | 4232.9226 | -0.0047 |
| 2 | 1 | 2 | 1 | 0 | 1 | 1 | 1 | 6675.1353 | 0.0002 |
| | | | | | | 2 | 1 | 6675.8315 | -0.0053 |
| | | | | | | 3 | 2 | 6675.9901 | 0.0017 |
| | | | | | | 2 | 2 | 6676.4354 | -0.0041 |
| | | | | | | 1 | 0 | 6676.6507 | 0.0088 |
| 2 | 1 | 1 | 1 | 0 | 1 | 1 | 1 | 7077.4645 | 0.0013 |
| | | | | | | 2 | 1 | 7077.7674 | 0.0017 |
| | | | | | | 3 | 2 | 7078.1724 | -0.0014 |
| | | | | | | 2 | 2 | 7078.3543 | -0.0141 |
| | | | | | | 1 | 0 | 7078.9753 | 0.0053 |
| 3 | 1 | 3 | 2 | 1 | 2 | 4 | 3 | 5943.4620 | -0.0044 |
| | | | | | | 3 | 2 | 5943.6561 | 0.0037 |
| 3 | 0 | 3 | 2 | 0 | 2 | 2 | 2 | 6127.3020 | 0.0012 |
| | | | | | | 4 | 3 | 6128.1983 | -0.0066 |
| | | | | | | 3 | 2 | 6128.1983 | -0.0066 |
| | | | | | | 2 | 1 | 6128.3124 | -0.0014 |
| | | | | | | 2 | 2 | 6127.3020 | 0.0012 |
| | | | | | | 3 | 3 | 6128.8802 | 0.0044 |
| 3 | 1 | 2 | 2 | 1 | 1 | 4 | 3 | 6345.4948 | 0.0033 |
| | | | | | | 3 | 2 | 6345.6656 | -0.0023 |
| | | | | | | 3 | 3 | 6345.8653 | 0.0028 |
| 3 | 2 | 2 | 2 | 2 | 1 | 2 | 1 | 6147.0567 | -0.0040 |
| | | | | | | 3 | 3 | 6147.4197 | 0.0003 |
| | | | | | | 4 | 3 | 6147.4197 | 0.0003 |
| | | | | | | 3 | 2 | 6148.0635 | -0.0018 |
| | | | | | | 2 | 2 | 6148.0635 | -0.0018 |
| 3 | 2 | 1 | 2 | 2 | 0 | 2 | 1 | 6166.4110 | -0.0002 |
| | | | | | | 3 | 3 | 6166.7654 | -0.0014 |
| | | | | | | 4 | 3 | 6166.7654 | -0.0014 |
| | | | | | | 3 | 2 | 6167.4022 | -0.0027 |
| 3 | 2 | 1 | 3 | 1 | 2 | 4 | 4 | 7961.2590 | 0.0030 |
| | | | | | | 2 | 2 | 7961.3885 | -0.0024 |

**Table S4.** Continuation.

| J´ | $K_{-1}$´ | $K_{+1}$´ | J´´ | $K_{-1}$´´ | $K_{+1}$´´ | F´ | F´´ | Obs. | Obs.-Cal. |
|---|---|---|---|---|---|---|---|---|---|
| 4 | 0 | 4 | 3 | 0 | 3 | 5 | 4 | 8148.6286 | 0.0056 |
| 4 | 1 | 4 | 3 | 1 | 3 | 5 | 4 | 7919.2541 | 0.0059 |
| | | | | | | 4 | 3 | 7919.3310 | -0.0028 |
| 4 | 0 | 4 | 3 | 1 | 3 | 5 | 4 | 5750.8520 | 0.0011 |
| | | | | | | 4 | 3 | 5750.9191 | -0.0059 |
| 4 | 0 | 4 | 3 | 1 | 2 | 5 | 4 | 4946.6458 | 0.0054 |
| | | | | | | 4 | 3 | 4946.9804 | -0.0003 |
| 4 | 2 | 2 | 4 | 1 | 3 | 4 | 4 | 7747.2800 | 0.0013 |
| | | | | | | 5 | 5 | 7747.4888 | 0.0079 |
| | | | | | | 3 | 3 | 7747.5388 | 0.0059 |
| 4 | 2 | 3 | 4 | 1 | 3 | 4 | 4 | 7675.0160 | 0.0009 |
| | | | | | | 5 | 5 | 7675.2060 | -0.0020 |
| | | | | | | 3 | 3 | 7675.2060 | -0.0020 |
| 5 | 0 | 5 | 4 | 1 | 4 | 6 | 5 | 7982.4025 | 0.0028 |
| | | | | | | 5 | 4 | 7982.4025 | -0.0111 |
| 5 | 2 | 3 | 5 | 1 | 4 | 5 | 5 | 7518.9196 | 0.0042 |
| | | | | | | 6 | 6 | 7519.0255 | -0.0070 |
| 5 | 2 | 4 | 5 | 1 | 4 | 5 | 5 | 7351.6069 | 0.0039 |
| | | | | | | 6 | 6 | 7351.6836 | -0.0015 |
| | | | | | | 4 | 4 | 7351.6836 | -0.0015 |
| | | | | | | 5 | 6 | 7352.0874 | -0.0123 |
| 6 | 1 | 5 | 6 | 0 | 6 | 6 | 6 | 4438.9496 | 0.0056 |
| | | | | | | 7 | 7 | 4439.1981 | 0.0037 |
| | | | | | | 5 | 5 | 4439.1981 | 0.0037 |
| 6 | 2 | 4 | 6 | 1 | 5 | 6 | 6 | 7299.9332 | 0.0014 |
| | | | | | | 7 | 7 | 7300.0159 | 0.0017 |
| | | | | | | 5 | 5 | 7300.0159 | 0.0017 |
| 7 | 1 | 6 | 7 | 0 | 7 | 7 | 7 | 5090.2479 | 0.0064 |
| | | | | | | 8 | 8 | 5090.4692 | -0.0068 |
| | | | | | | 6 | 6 | 5090.5057 | -0.0041 |
| 7 | 2 | 6 | 7 | 1 | 6 | 6 | 6 | 6533.6060 | -0.0010 |
| | | | | | | 8 | 8 | 6533.6060 | -0.0010 |
| | | | | | | 7 | 7 | 6533.6060 | -0.0010 |
| 7 | 2 | 5 | 7 | 1 | 6 | 7 | 7 | 7116.4568 | -0.0025 |
| | | | | | | 8 | 8 | 7116.5241 | 0.0044 |
| | | | | | | 6 | 6 | 7116.5241 | -0.0042 |
| 8 | 2 | 6 | 8 | 1 | 7 | 7 | 7 | 6049.5121 | 0.0055 |
| | | | | | | 9 | 9 | 6049.5121 | 0.0055 |
| 8 | 2 | 6 | 8 | 1 | 7 | 8 | 8 | 6994.5214 | -0.0013 |
| | | | | | | 9 | 9 | 6994.5794 | -0.0036 |
| | | | | | | 7 | 7 | 6994.5794 | -0.0036 |
| 9 | 2 | 8 | 9 | 1 | 8 | 8 | 8 | 5525.3848 | -0.0015 |
| | | | | | | 10 | 10 | 5525.3848 | -0.0015 |
| 9 | 2 | 7 | 9 | 1 | 8 | 9 | 9 | 6958.1350 | -0.0013 |
| | | | | | | 10 | 10 | 6958.2025 | 0.0016 |
| | | | | | | 8 | 8 | 6958.2025 | 0.0016 |

**Table S4.** Continuation.

| J´ | $K_{-1}$´ | $K_{+1}$´ | J´´ | $K_{-1}$´´ | $K_{+1}$´´ | F´ | F´´ | Obs. | Obs.-Cal. |
|---|---|---|---|---|---|---|---|---|---|
| 10 | 2 | 8 | 10 | 1 | 9 | 10 | 10 | 7028.2098 | 0.0039 |
| | | | | | | 11 | 11 | 7028.2774 | -0.0021 |
| | | | | | | 9 | 9 | 7028.2774 | -0.0021 |

**Table S5.** Observed rotational transitions and residuals (all the values in MHz) for the II-ax-ta SPro conformer in the ground vibrational state.

| J′ | $K_{-1}$′ | $K_{+1}$′ | J′′ | $K_{-1}$′′ | $K_{+1}$′′ | F′ | F′′ | Obs. | Obs.-Cal. |
|---|---|---|---|---|---|---|---|---|---|
| 1 | 1 | 1 | 0 | 0 | 0 | 1 | 1 | 4305.3583 | -0.0033 |
| | | | | | | 2 | 1 | 4305.5233 | -0.0001 |
| | | | | | | 0 | 1 | 4305.7641 | -0.0018 |
| 1 | 1 | 0 | 0 | 0 | 0 | 0 | 1 | 4532.4988 | 0.0000 |
| | | | | | | 2 | 1 | 4533.3356 | 0.0003 |
| | | | | | | 1 | 1 | 4533.8895 | -0.0033 |
| 2 | 1 | 2 | 1 | 1 | 1 | 2 | 2 | 4586.1557 | -0.0011 |
| | | | | | | 2 | 1 | 4586.3182 | -0.0004 |
| | | | | | | 3 | 2 | 4586.7551 | 0.0007 |
| | | | | | | 1 | 0 | 4586.8429 | -0.0007 |
| | | | | | | 1 | 1 | 4587.2511 | 0.0031 |
| 2 | 0 | 2 | 1 | 0 | 1 | 2 | 2 | 4794.7937 | 0.0004 |
| | | | | | | 1 | 0 | 4794.9276 | 0.0039 |
| | | | | | | 2 | 1 | 4795.1875 | -0.0016 |
| | | | | | | 3 | 2 | 4795.2597 | 0.0008 |
| | | | | | | 1 | 1 | 4795.9154 | 0.0019 |
| 2 | 1 | 1 | 1 | 1 | 0 | 1 | 1 | 5041.9130 | 0.0016 |
| | | | | | | 2 | 1 | 5042.1827 | 0.0020 |
| | | | | | | 3 | 2 | 5042.5649 | -0.0001 |
| | | | | | | 2 | 2 | 5042.7389 | 0.0006 |
| | | | | | | 1 | 0 | 5043.3080 | 0.0026 |
| 2 | 2 | 1 | 2 | 1 | 1 | 1 | 2 | 5694.0095 | 0.0019 |
| | | | | | | 1 | 1 | 5694.2771 | 0.0002 |
| | | | | | | 3 | 3 | 5694.4180 | 0.0015 |
| | | | | | | 2 | 2 | 5694.6682 | 0.0008 |
| | | | | | | 2 | 3 | 5694.8404 | -0.0002 |
| | | | | | | 2 | 1 | 5694.9311 | -0.0056 |
| 2 | 0 | 2 | 2 | 1 | 1 | 1 | 2 | 5713.3041 | 0.0106 |
| | | | | | | 3 | 2 | 5713.5667 | 0.0067 |
| | | | | | | 1 | 1 | 5713.5667 | 0.0067 |
| | | | | | | 3 | 3 | 5713.7283 | 0.0029 |
| | | | | | | 2 | 2 | 5714.0189 | 0.0012 |
| | | | | | | 2 | 3 | 5714.1923 | 0.0013 |
| | | | | | | 2 | 1 | 5714.2920 | 0.0049 |
| 2 | 2 | 1 | 2 | 1 | 2 | 1 | 1 | 6377.4665 | -0.0049 |
| | | | | | | 3 | 3 | 6378.0384 | -0.0005 |
| | | | | | | 2 | 2 | 6379.0608 | 0.0001 |
| 2 | 2 | 0 | 2 | 1 | 2 | 1 | 1 | 6396.7537 | -0.0036 |
| | | | | | | 3 | 3 | 6397.3485 | 0.0005 |
| | | | | | | 2 | 2 | 6398.4124 | 0.0014 |
| 2 | 1 | 2 | 1 | 0 | 1 | 2 | 2 | 6484.3298 | 0.0023 |
| | | | | | | 1 | 0 | 6484.6614 | -0.0016 |
| | | | | | | 2 | 1 | 6484.7209 | -0.0024 |
| | | | | | | 3 | 2 | 6484.9235 | -0.0014 |
| | | | | | | 1 | 1 | 6485.6499 | -0.0028 |

**Table S5.** Continuation.

| J′ | K-1′ | K+1′ | J′′ | K-1′′ | K+1′′ | F′ | F′′ | Obs. | Obs.-Cal. |
|---|---|---|---|---|---|---|---|---|---|
| 2 | 1 | 1 | 1 | 0 | 1 | 1 | 0 | 7167.8534 | -0.0041 |
| | | | | | | 3 | 2 | 7168.5448 | -0.0027 |
| | | | | | | 2 | 2 | 7168.7206 | -0.0001 |
| | | | | | | 1 | 1 | 7168.8455 | -0.0018 |
| | | | | | | 2 | 1 | 7169.1146 | -0.0021 |
| 3 | 1 | 3 | 2 | 1 | 2 | 3 | 3 | 6867.5935 | -0.0045 |
| | | | | | | 3 | 2 | 6868.1954 | -0.0001 |
| | | | | | | 2 | 1 | 6868.2682 | 0.0000 |
| | | | | | | 4 | 3 | 6868.3401 | -0.0001 |
| | | | | | | 2 | 2 | 6869.2007 | 0.0032 |
| 3 | 0 | 3 | 2 | 0 | 2 | 3 | 3 | 7144.8502 | 0.0019 |
| | | | | | | 3 | 2 | 7145.3155 | -0.0031 |
| | | | | | | 2 | 1 | 7145.3155 | -0.0031 |
| | | | | | | 4 | 3 | 7145.3934 | -0.0001 |
| | | | | | | 2 | 2 | 7146.0415 | -0.0085 |
| 3 | 2 | 2 | 2 | 2 | 1 | 3 | 2 | 7221.6346 | 0.0001 |
| | | | | | | 2 | 2 | 7221.6346 | 0.0001 |
| | | | | | | 3 | 3 | 7222.0594 | 0.0007 |
| | | | | | | 4 | 3 | 7222.0594 | 0.0007 |
| | | | | | | 2 | 3 | 7222.0594 | 0.0007 |
| | | | | | | 2 | 1 | 7222.2952 | 0.0008 |
| 3 | 2 | 1 | 2 | 2 | 0 | 2 | 2 | 7297.9002 | -0.0024 |
| | | | | | | 3 | 2 | 7298.0445 | -0.0003 |
| | | | | | | 4 | 3 | 7298.4049 | -0.0001 |
| | | | | | | 3 | 3 | 7298.5042 | -0.0063 |
| | | | | | | 2 | 1 | 7298.6245 | -0.0023 |
| 3 | 1 | 2 | 2 | 1 | 1 | 2 | 2 | 7550.8821 | 0.0002 |
| | | | | | | 3 | 2 | 7550.9585 | 0.0020 |
| | | | | | | 4 | 3 | 7551.0741 | -0.0002 |
| | | | | | | 2 | 1 | 7551.1547 | 0.0035 |
| 3 | 0 | 3 | 2 | 1 | 1 | 3 | 2 | 4771.3864 | 0.0001 |
| | | | | | | 4 | 3 | 4772.1081 | 0.0032 |
| | | | | | | 2 | 1 | 4772.3930 | 0.0011 |
| 3 | 0 | 3 | 2 | 1 | 2 | 3 | 3 | 5455.1828 | 0.0007 |
| | | | | | | 2 | 1 | 5455.5878 | 0.0014 |
| | | | | | | 4 | 3 | 5455.7343 | -0.0144 |
| | | | | | | 3 | 2 | 5455.7343 | -0.0144 |
| | | | | | | 2 | 2 | 5456.5165 | 0.0007 |
| 3 | 2 | 1 | 3 | 1 | 2 | 2 | 2 | 5461.0810 | 0.0134 |
| | | | | | | 4 | 4 | 5461.0810 | 0.0134 |
| | | | | | | 3 | 3 | 5461.0810 | 0.0134 |
| 3 | 1 | 3 | 2 | 0 | 2 | 4 | 3 | 8558.0086 | 0.0018 |
| | | | | | | 2 | 1 | 8558.0086 | 0.0018 |
| 3 | 2 | 2 | 3 | 1 | 2 | 3 | 3 | 5365.3516 | 0.0061 |
| | | | | | | 3 | 4 | 5365.3869 | -0.0138 |
| | | | | | | 4 | 4 | 5365.3869 | -0.0138 |

**Table S5.** Continuation.

| J′ | K-1′ | K+1′ | J′′ | K-1′′ | K+1′′ | F′ | F′′ | Obs. | Obs.-Cal. |
|---|---|---|---|---|---|---|---|---|---|
| 3 | 2 | 2 | 3 | 1 | 3 | 3 | 2 | 6731.4922 | -0.0055 |
| | | | | | | 2 | 2 | 6731.4922 | -0.0055 |
| | | | | | | 3 | 4 | 6731.7582 | 0.0009 |
| | | | | | | 4 | 4 | 6731.7582 | 0.0009 |
| | | | | | | 3 | 3 | 6732.4983 | -0.0013 |
| | | | | | | 4 | 3 | 6732.4983 | -0.0013 |
| | | | | | | 2 | 3 | 6732.4983 | -0.0013 |
| 3 | 2 | 1 | 3 | 1 | 3 | 2 | 2 | 6827.1129 | -0.0031 |
| | | | | | | 4 | 4 | 6827.4151 | 0.0024 |
| | | | | | | 3 | 3 | 6828.2572 | -0.0031 |
| 4 | 2 | 2 | 4 | 1 | 3 | 3 | 3 | 5217.5684 | -0.0014 |
| | | | | | | 5 | 5 | 5217.5684 | -0.0014 |
| | | | | | | 4 | 4 | 5217.5684 | -0.0014 |
| 4 | 1 | 3 | 3 | 2 | 2 | 4 | 3 | 4677.4858 | 0.0034 |
| | | | | | | 4 | 4 | 4677.4858 | 0.0034 |
| | | | | | | 5 | 4 | 4677.4858 | 0.0034 |
| | | | | | | 3 | 2 | 4677.4858 | 0.0034 |
| | | | | | | 3 | 3 | 4677.4858 | 0.0034 |
| 4 | 1 | 3 | 3 | 2 | 1 | 4 | 3 | 4581.7114 | -0.0013 |
| | | | | | | 5 | 4 | 4581.8371 | 0.0066 |
| 4 | 2 | 3 | 4 | 1 | 3 | 4 | 4 | 4936.4642 | 0.0025 |
| | | | | | | 5 | 5 | 4936.6485 | 0.0087 |
| | | | | | | 3 | 3 | 4936.6832 | -0.0022 |
| 4 | 2 | 3 | 4 | 1 | 4 | 4 | 3 | 7208.1638 | 0.0087 |
| | | | | | | 3 | 3 | 7208.3902 | -0.0040 |
| | | | | | | 5 | 5 | 7208.5557 | -0.0010 |
| | | | | | | 4 | 4 | 7209.1886 | -0.0003 |
| 4 | 0 | 4 | 3 | 1 | 2 | 4 | 3 | 6663.8379 | 0.0003 |
| | | | | | | 5 | 4 | 6664.5323 | 0.0030 |
| | | | | | | 3 | 2 | 6664.7120 | -0.0001 |
| 4 | 2 | 2 | 4 | 1 | 4 | 4 | 3 | 7489.2684 | -0.0056 |
| | | | | | | 3 | 3 | 7489.2684 | -0.0056 |
| | | | | | | 4 | 5 | 7489.4817 | -0.0029 |
| | | | | | | 5 | 5 | 7489.4817 | -0.0029 |
| | | | | | | 4 | 4 | 7490.3049 | 0.0012 |
| | | | | | | 5 | 4 | 7490.3049 | 0.0012 |
| | | | | | | 3 | 4 | 7490.3049 | 0.0012 |
| 5 | 1 | 4 | 5 | 0 | 5 | 4 | 4 | 4202.8698 | -0.0041 |
| | | | | | | 6 | 6 | 4203.0106 | 0.0017 |
| | | | | | | 5 | 5 | 4203.6690 | -0.0023 |
| 5 | 2 | 3 | 5 | 1 | 4 | 4 | 4 | 5050.9388 | -0.0043 |
| | | | | | | 6 | 6 | 5050.9388 | -0.0043 |
| | | | | | | 5 | 5 | 5050.9388 | -0.0043 |
| 5 | 2 | 4 | 5 | 1 | 5 | 4 | 4 | 7810.0995 | 0.0014 |
| | | | | | | 6 | 6 | 7810.2131 | -0.0028 |
| | | | | | | 5 | 5 | 7810.7957 | 0.0009 |

**Table S5.** Continuation.

| J´ | $K_{-1}$´ | $K_{+1}$´ | J´´ | $K_{-1}$´´ | $K_{+1}$´´ | F´ | F´´ | Obs. | Obs.-Cal. |
|---|---|---|---|---|---|---|---|---|---|
| 5 | 1 | 4 | 4 | 2 | 3 | 6 | 5 | 7573.3518 | 0.0054 |
| | | | | | | 5 | 4 | 7573.4716 | 0.0039 |
| 5 | 1 | 5 | 4 | 2 | 3 | 5 | 4 | 4182.3736 | -0.0069 |
| | | | | | | 6 | 5 | 4183.0705 | 0.0006 |
| 5 | 2 | 4 | 5 | 1 | 4 | 5 | 5 | 4419.7061 | -0.0014 |
| | | | | | | 6 | 6 | 4419.9467 | 0.0072 |
| | | | | | | 4 | 4 | 4419.9869 | 0.0002 |
| 5 | 1 | 4 | 4 | 2 | 2 | 5 | 5 | 7292.3556 | 0.0023 |
| | | | | | | 5 | 4 | 7292.3556 | 0.0023 |
| | | | | | | 6 | 5 | 7292.4266 | 0.0030 |
| | | | | | | 4 | 3 | 7292.4266 | 0.0030 |
| 6 | 2 | 4 | 6 | 1 | 5 | 7 | 7 | 5025.6173 | 0.0056 |
| | | | | | | 6 | 6 | 5025.7164 | -0.0025 |
| 6 | 1 | 6 | 5 | 2 | 3 | 7 | 6 | 5189.4462 | 0.0052 |
| | | | | | | 5 | 4 | 5189.5749 | -0.0170 |
| 6 | 1 | 5 | 6 | 0 | 6 | 5 | 5 | 5265.1572 | -0.0026 |
| | | | | | | 7 | 7 | 5265.2776 | 0.0008 |
| | | | | | | 6 | 6 | 5265.9709 | 0.0010 |
| 6 | 2 | 4 | 5 | 3 | 3 | 6 | 5 | 5325.8356 | 0.0052 |
| | | | | | | 7 | 6 | 5325.8356 | 0.0052 |
| | | | | | | 5 | 4 | 5325.8356 | 0.0052 |
| | | | | | | 5 | 5 | 5325.8356 | 0.0052 |
| 6 | 1 | 6 | 5 | 2 | 4 | 6 | 5 | 5819.8114 | 0.0034 |
| | | | | | | 7 | 6 | 5820.4402 | 0.0070 |
| | | | | | | 5 | 4 | 5820.5323 | 0.0040 |
| 7 | 2 | 5 | 7 | 1 | 6 | 8 | 8 | 5194.6485 | 0.0057 |
| | | | | | | 7 | 7 | 5194.8235 | -0.0023 |
| 7 | 2 | 6 | 6 | 3 | 3 | 8 | 7 | 6227.9873 | -0.0009 |
| | | | | | | 6 | 5 | 6228.0374 | -0.0025 |
| 7 | 1 | 6 | 7 | 0 | 7 | 6 | 6 | 6547.6590 | 0.0014 |
| | | | | | | 8 | 8 | 6547.7545 | -0.0044 |
| | | | | | | 7 | 7 | 6548.4631 | 0.0009 |
| 7 | 2 | 6 | 6 | 3 | 4 | 7 | 6 | 6311.7203 | -0.0007 |
| | | | | | | 8 | 7 | 6312.0516 | 0.0007 |
| 7 | 1 | 7 | 6 | 2 | 5 | 8 | 7 | 7319.0498 | 0.0018 |
| | | | | | | 6 | 5 | 7319.1165 | -0.0069 |
| 8 | 2 | 6 | 8 | 1 | 7 | 7 | 7 | 5598.9190 | 0.0120 |
| | | | | | | 9 | 9 | 5598.9190 | 0.0120 |
| | | | | | | 8 | 8 | 5599.1795 | -0.0018 |
| 8 | 3 | 6 | 8 | 2 | 6 | 9 | 9 | 7967.9561 | -0.0060 |
| 9 | 2 | 7 | 9 | 1 | 8 | 8 | 8 | 6268.2780 | -0.0087 |
| | | | | | | 10 | 10 | 6268.2780 | -0.0087 |
| | | | | | | 9 | 9 | 6268.6380 | 0.0021 |
| 9 | 3 | 6 | 9 | 2 | 7 | 9 | 9 | 7993.3695 | 0.0052 |
| | | | | | | 10 | 10 | 7993.4425 | 0.0047 |
| | | | | | | 8 | 8 | 7993.4425 | 0.0047 |

**Table S5.** Continuation.

| J′ | $K_{-1}$′ | $K_{+1}$′ | J′′ | $K_{-1}$′′ | $K_{+1}$′′ | F′ | F′′ | Obs. | Obs.-Cal. |
|---|---|---|---|---|---|---|---|---|---|
| 9 | 3 | 7 | 9 | 2 | 7 | 9 | 9 | 7166.1897 | 0.0025 |
| | | | | | | 10 | 10 | 7166.4322 | -0.0013 |
| 10 | 2 | 8 | 10 | 1 | 9 | 9 | 9 | 7217.3855 | 0.0028 |
| | | | | | | 11 | 11 | 7217.3855 | 0.0028 |
| | | | | | | 10 | 10 | 7217.7946 | -0.0012 |
| 10 | 3 | 7 | 10 | 2 | 8 | 10 | 10 | 7707.9686 | -0.0020 |
| | | | | | | 11 | 11 | 7707.9686 | -0.0020 |
| | | | | | | 9 | 9 | 7707.9686 | -0.0020 |
| 10 | 3 | 7 | 10 | 2 | 8 | 10 | 10 | 7707.9686 | -0.0020 |
| | | | | | | 11 | 11 | 7707.9686 | -0.0020 |
| | | | | | | 9 | 9 | 7707.9686 | -0.0020 |
| 11 | 3 | 8 | 11 | 2 | 9 | 10 | 10 | 7620.0436 | 0.0012 |
| | | | | | | 12 | 12 | 7620.0436 | 0.0012 |
| 12 | 3 | 9 | 12 | 2 | 10 | 11 | 11 | 7786.6612 | -0.0028 |
| | | | | | | 13 | 13 | 7786.6612 | -0.0028 |
| | | | | | | 12 | 12 | 7786.7822 | -0.0018 |
